# Supporting women of childbearing age in the prevention and treatment of overweight and obesity: a scoping review of randomized control trials of behavioral interventions

**DOI:** 10.1186/s12905-020-0882-3

**Published:** 2020-01-23

**Authors:** Melinda J. Hutchesson, Mette de Jonge Mulock Houwer, Hannah M. Brown, Siew Lim, Lisa J. Moran, Lisa Vincze, Megan E. Rollo, Jenna L. Hollis

**Affiliations:** 1 School of Health Sciences, Faculty of Health and Medicine, and Priority Research Centre for Physical Activity and Nutrition, University of Newcastle, University Drive, Callaghan, New South Wales Australia; 2 Division of Human Nutrition and Health, Wageningen University, Wageningen, The Netherlands; 3 Monash Centre for Health Research and Implementation, Monash University, Clayton, Victoria Australia; 4 School of Allied Health Science, Griffith University, Gold Coast, Queensland Australia; 5 Hunter New England Population Health, Longworth Avenue, Wallsend, Australia; 6 School of Medicine and Public Health, The University of Newcastle, Callaghan, Australia; 7 Priority Research Centre for Health Behaviour, University of Newcastle, Callaghan, New South Wales Australia; 8 Hunter Medical Research Institute, New Lambton Heights, NSW Australia

**Keywords:** Childbearing, Women, Obesity treatment, Obesity prevention, Scoping review

## Abstract

**Background:**

Women of childbearing age are vulnerable to weight gain. This scoping review examines the extent and range of research undertaken to evaluate behavioral interventions to support women of childbearing age to prevent and treat overweight and obesity.

**Methods:**

Eight electronic databases were searched for randomized controlled trials (RCT) or systematic reviews of RCTs until 31st January 2018. Eligible studies included women of childbearing age (aged 15–44 years), evaluated interventions promoting behavior change related to diet or physical activity to achieve weight gain prevention, weight loss or maintenance and reported weight-related outcomes.

**Results:**

Ninety studies met the inclusion criteria (87 RCTs, 3 systematic reviews). Included studies were published from 1998 to 2018. The studies primarily focused on preventing excessive gestational weight gain (*n* = 46 RCTs, *n* = 2 systematic reviews), preventing postpartum weight retention (*n* = 18 RCTs) or a combination of the two (*n* = 14 RCTs, *n* = 1 systematic review). The RCTs predominantly evaluated interventions that aimed to change both diet and physical activity behaviors (*n* = 84) and were delivered in-person (*n* = 85).

**Conclusions:**

This scoping review identified an increasing volume of research over time undertaken to support women of childbearing age to prevent and treat overweight and obesity. It highlights, however, that little research is being undertaken to support the young adult female population unrelated to pregnancy or preconception.

## Background

Prevalence of women affected by obesity is increasing globally, with prevalence rates increasing from 6.4% in 1975 to 14.9% in 2014 [1]. Women of childbearing age (15 to 44 years) are particularly vulnerable to weight gain, with many large cohort studies demonstrating this life stage is the time of greatest weight gain [2–5]. For example, the Australian Longitudinal Study of Women’s Health has found women in their younger cohort (aged 18–23 years at survey 1) experience an average weight gain of 6.3 kg over 10 years [3]. Notably, in women of childbearing age, pregnancy has been investigated as a potential trigger for excessive weight gain and the development of overweight and obesity. The results however are inconsistent, with some studies in women of childbearing age demonstrating an association between parity and weight gain and/or the development of overweight and obesity, while others have shown no association [6, 7].

Weight gained during the childbearing years is strongly associated with adverse health outcomes later in life. For example, the Nurses’ Health Study (*n* = 92,837) identified that for every additional 5 kg of weight gained from 21 years of age there were 142.6 additional cases of Type 2 diabetes (per 100,000 person-years from age 55 onwards), 458.8 for hypertension, 36.9 for cardiovascular disease, 36.9 for cancer, and 76.7 for overall mortality [8]. Furthermore, obesity during the childbearing years has been associated with adverse pregnancy outcomes for the mother (e.g. gestational diabetes, pre-eclampsia, gestational hypertension, antenatal anxiety and postpartum depression), as well as for the babies (e.g. pre-term birth, and large for gestational age babies) [9, 10].

Behavioral interventions to support women to prevent weight gain during the childbearing years, or treat overweight or obesity, have the potential to have an impact on the health and well-being of women, as well as their offspring. Behavioral interventions are those designed to influence individuals’ actions, more specifically for weight management, interventions include those which influence physical activity and sedentary and/or dietary behaviors. A large number of systematic reviews have been undertaken to determine the most effective interventions to support women during this life stage to prevent and treat overweight and obesity [11–23]. However, reviews published to date have typically been limited to one type of intervention (e.g. treatment or prevention of obesity, preventing excessive gestational weight gain (GWG)), population group (e.g. pregnant/postpartum women) or mode of delivery (e.g. web-based), or combined a variety of intervention approaches (e.g. behavioral, surgical, pharmacological) in the one review. Therefore, previous systematic reviews are unable to determine the most appropriate time for intervention during this life stage (e.g. pre-conception, postpartum), the most efficacious behavioral intervention approach (e.g. treatment, prevention) nor the optimal mode of delivery for intervention.

To our knowledge, no single review has identified all behavioral interventions aiming to support women of childbearing age to prevent weight gain, or achieve weight loss or weight loss maintenance. Therefore, the aim of this scoping review is to examine the extent and range of research undertaken to evaluate behavioral interventions that support women of childbearing age to prevent or treat overweight and obesity. The scoping review methodology allows mapping of the range of research that has been undertaken over time. The scoping review is the first step in determining the most efficacious intervention approach. It will help identify gaps in research undertaken to date, and determine whether a full systematic review can be undertaken.

## Methods

A scoping review was undertaken using a predefined protocol following the methodological framework of Arksey and O’Malley, [24] including identifying the research question, searching for relevant studies, selecting studies, charting the data and collating, summarizing, and reporting the results. The conduct and reporting of the scoping review is consistent with the Preferred Reporting Items for Systematic reviews and Meta-Analyses extension for Scoping Reviews (PRISMA-ScR) Checklist. Preliminary findings of the scoping review were previously presented [25].

### Identifying the research question

The research question was operationalized using the Population-Intervention-Comparison-Outcome-Study design (PICOS) format. Inclusion criteria for the scoping review were therefore as follows:

#### Participants

Women of childbearing age (i.e. aged 15 to 44 years as per US Centre for Disease Control Definition).

#### Interventions

Interventions promoting behavior change (e.g. dietary behavior, physical activity and/or sedentary behavior) to prevent weight gain or overweight and obesity, or achieve weight loss or weight loss maintenance were included. Non-behavioral interventions, including very low energy diets (including meal replacements), weight loss medications and surgery alone or in combination with behavioral interventions will be excluded.

#### Comparators

No intervention control group, wait-list control group, standard/usual care or another active behavioral intervention.

#### Outcomes

To be included in the review studies must have measured and reported weight-related outcomes (e.g. weight, BMI, percentage body fat, waist circumference).

#### Study design

Systematic reviews of randomized control trials (RCTs) and RCTs as the two highest levels of evidence for evaluating interventions [26].

### Selection of studies relevant to the research question

#### Search strategy

The search strategy, including database selection and search teams, was developed in consultation with an expert medical librarian. The search aimed to find peer reviewed journal articles published in English. All sources were searched from the date of inception up until the 31st January 2018 (Additional file 1: Table S1). The databases searched were MEDLINE (Ovid), MEDLINE in process (Ovid), EMBASE (Ovid), PsycINFO (Ovid), Scopus, CINAHL (EbscoHost) and Cochrane Library (Wiley). The reference lists of all included articles and reports were also searched for additional studies.

#### Screening

Screening was managed using Covidence (www.covidence.org). Title, abstract and keywords of all identified papers were each assessed by two independent reviewers (Reviewer 1: MMH; Reviewer 2: MH, JG, SL, LM, LV). Full text screening was conducted by two independent reviewers, and reasons for exclusions recorded (MMH, MH). A third reviewer was consulted for all conflicts for both abstract and full text screening (MR).

### Charting of information and data within the included studies

Data were extracted by one reviewer (MMH) and checked by a second reviewer (LV, LM, SL, JH, MR). Any differences between the first and second extractor were resolved by a third reviewer (HB). Data extracted included: study characteristics (i.e. year study conducted/published, country of origin, study design, number of study arms and comparators, intervention duration); participants (i.e. study inclusion criteria relating to age, BMI, ethnicity, socio-economic status, parity, clinical conditions or pregnancy); interventions (i.e. goal [weight loss, weight loss maintenance, weight gain prevention, excessive gestational weight gain prevention], behavior change promoted [dietary behavior, physical activity and/or sedentary behavior], setting [e.g. clinic, community], mode of delivery [i.e. individual, group or combination] and medium of delivery [e.g. in-person, website] and profession of intervention deliverer) and outcomes (i.e. what weight-related outcomes were measured and when, and other outcomes measured).

### Collating, summarizing and reporting results of the review

As is convention in scoping reviews, a numerical analysis was undertaken to elucidate the number of the studies, as well as changes overtime (based on publication date). In addition, results are presented by intervention goal, with studies group as general weight loss, post-partum weight loss/preventing weight retention, general weight gain prevention, excessive GWG, and combine excessive GWG prevention and post-partum weight loss. ‘General’ weight loss and weigh gain prevention includes those interventions unrelated to pregnancy status.

## Results

**Fig. 1:**
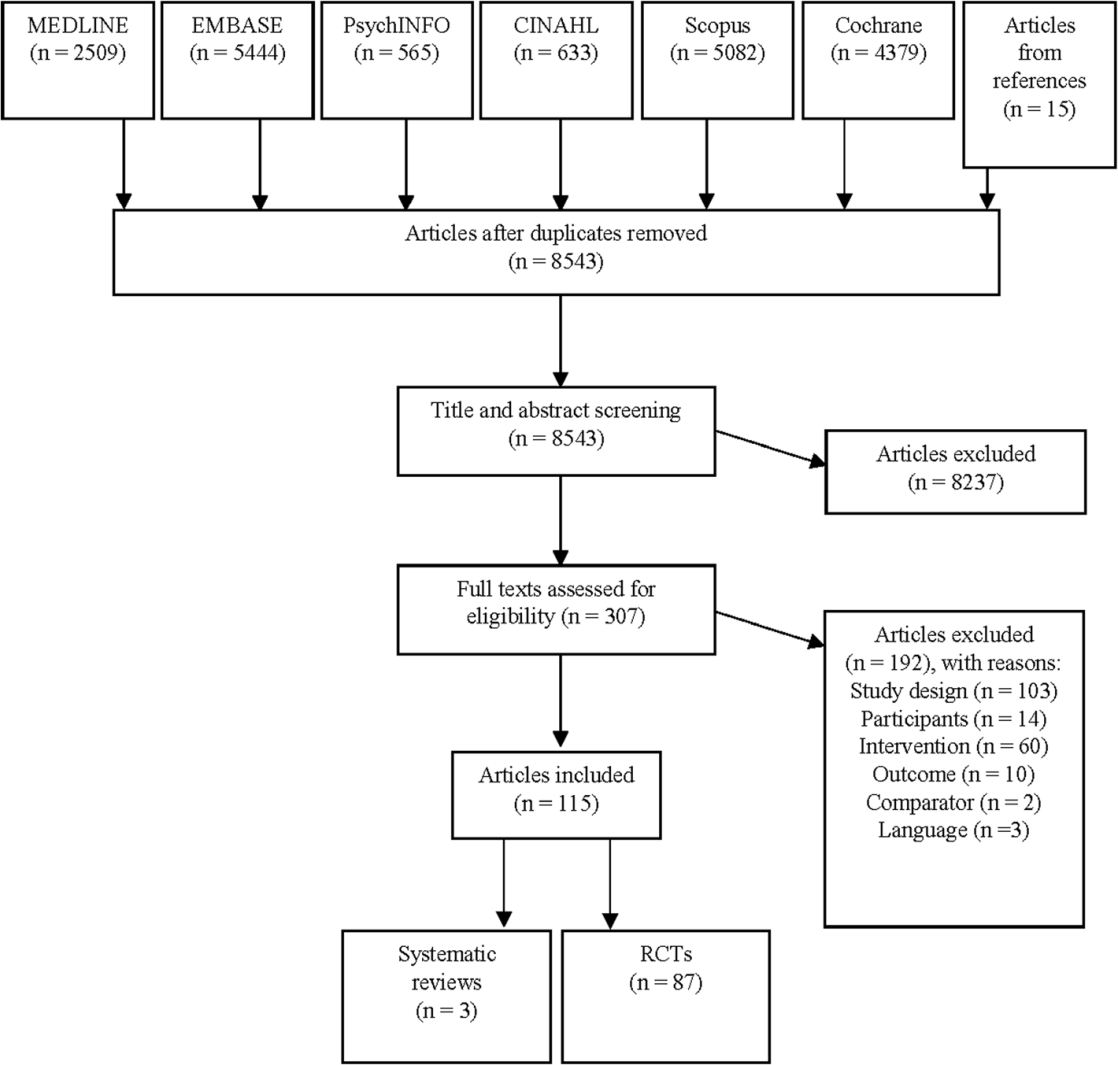
Flow diagram of included studies

Of the 8543 articles identified, 307 full text articles were assessed for eligibility and 115 articles met the inclusion criteria (Fig. 1). The articles described 87 RCTs and three systematic reviews.

### Randomized control trials

Table 1 describes the study characteristics of the included RCTs overall, and by intervention goal. Individual study characteristics are described in Additional file 1: Table S2. Of the 87 included RCTs, 52.9% (*n* = 46) focused on preventing excessive GWG, [27–71] 20.7% (*n* = 18) on weight loss or preventing weight retention in the postpartum period, [72–89] and 16.1% (*n* = 14) focused on both preventing excessive GWG and preventing weight retention in the postpartum period [90–103]. Few studies supported general reproductive-aged women (i.e. not specifically related to current or recent pregnancy) in weight loss (4.6%, *n* = 4) [104–107] or weight gain prevention (5.7%, *n* = 5) [108–112]. Most of the included studies were conducted in the United States (46.0%, *n* = 40), followed by Australia (16.1%, *n* = 14).

**Table 1:** Summary of study characteristics by intervention goal and in total

		Total (*n* = 87)	General weight loss (*n* = 4)	Postpartum weight loss/ preventing weight retention (*n* = 18)	General weight gain prevention (*n* = 5)	Excessive GWG prevention (*n* = 46)	Combined excessive GWG prevention & postpartum weight loss (*n* = 14)
Publication year	2000–2010, *n (%)*	17 (19.5)	2 (50)	4 (22.2)	2 (40)	8 (17.4)	1 (7.1)
	2011 - Jan 2018, *n (%)*	70 (80.5)	2 (50)	14 (77.8)	3 (60)	38 (82.6)	13 (92.9)
Country	United States, n (%)	40 (46.0)	2 (50)	14 (77.8)	4 (80)	12 (26.1)	8 (57.1)
	Australia, *n (%)*	14 (16.1)	1 (25)	1 (5.6)	–	11 (23.9)	1 (7.1)
	Sweden, *n (%)*	5 (5.8)	–	2 (11.1)	1 (20)	2 (4.4)	–
	Canada, *n (%)*	4 (4.6)	–	–	–	4 (8.7)	–
	Denmark, *n (%)*	3 (3.5)	–	–	–	3 (6.5)	–
	Other, *n (%)*	21 (24.1)	1 (25)	1 (5.6)	–	14 (30.4)	5 (35.7)
Number of participants	Total	26, 750	976	3878	586	15,929	5381
	Mean ± SD	307.5 ± 459.7	244 ± 198.2	215.4 ± 313.7	117.2 ± 65.9	346.3 ± 502.6	384.4 ± 555.1
	Median	150	166	82.5	102	197.5	237.5
	Range	16–2500	67–577	18–1325	40–194	16–2500	36–2280
Participant inclusion criteria: Age	Broad range- all childbearing women, *n (%)*	54 (62.1)	–	12 (66.7)	1 (20)	29 (63.0)	12 (85.7)
	Younger age groups (< 30 years), *n (%)*	5 (5.8)	1 (25)	1 (5.6)	1 (20)	1 (2.2)	1 (7.1)
	Specific/limited range, *n (%)*	12 (13.8)	3 (75)	2 (11.1)	3 (60)	2 (4.4)	–
	Age range not reported, *n (%)*	18 (20.7)	–	3 (16.7)	–	14 (30.4)	1 (7.1)
Participant inclusion criteria: BMI^a^	Underweight, healthy weight, overweight and obese*, n (%)*	2 (2.3)	–	–	–	2 (4.4)	–
	Underweight, healthy weight and overweight, *n (%)*	1 (1.2)	–	–	–	1 (2.2)	–
	Healthy weight and overweight, n (%)	1 (1.2)	–	–	1 (20)	–	–
	Healthy weight, overweight and obese, *n (%)*	16 (18.4)	–	3 (16.7)	1 (20)	10 (21.7)	2 (14.3)
	Overweight only, *n (%)*	1 (1.2)	–	1 (5.6)	–	–	–
	Overweight and obese, *n (%)*	36 (41.4)	4 (100)	12 (66.7)	3 (60)	13 (28.3)	4 (28.6)
	Obese only, *n (%)*	9 (10.3)	–	–	–	7 (15.2)	2 (14.3)
	Not reported, *n (%)*	21 (24.1)		2 (11.1)		13 (28.3)	6 (42.9)
Participant inclusion criteria: Pregnancy related	Antenatal, *n (%)*	59 (67.8)	–	–	–	45 (97.8)	14 (100)
	Postpartum, *n (%)*	18 (20.7)	–	18 (100)	–	–	–
	Planning pregnancy, *n (%)*	1 (1.15)	–	–	–	1 (2.2)	–
	Not pregnancy related, *n (%)*	9 (10.3)	4 (100)	–	5 (100)	–	–
Participant inclusion criteria: Other	Parity, *n (%)*	5 (5.8)	–	–	–	3 (6.5)	2 (14.3)
	Ethnicity, *n (%)*	12 (13.8)	1 (25)	2 (11.1)	3 (60)	3 (6.5)	3 (21.4)
	Socio-economic status, *n (%)*	10 (11.5)	1 (25)	4 (22.2)	1 (20)	3 (6.5)	1 (7.1)
	Clinical condition, *n (%)*	5 (5.8)	1 (25)	2 (11.1)	–	–	2 (14.3)

^a^BMI classifications as per the World Health Organization International Classification of adult underweight, overweight and obesity according to BMI

**Fig. 2:**
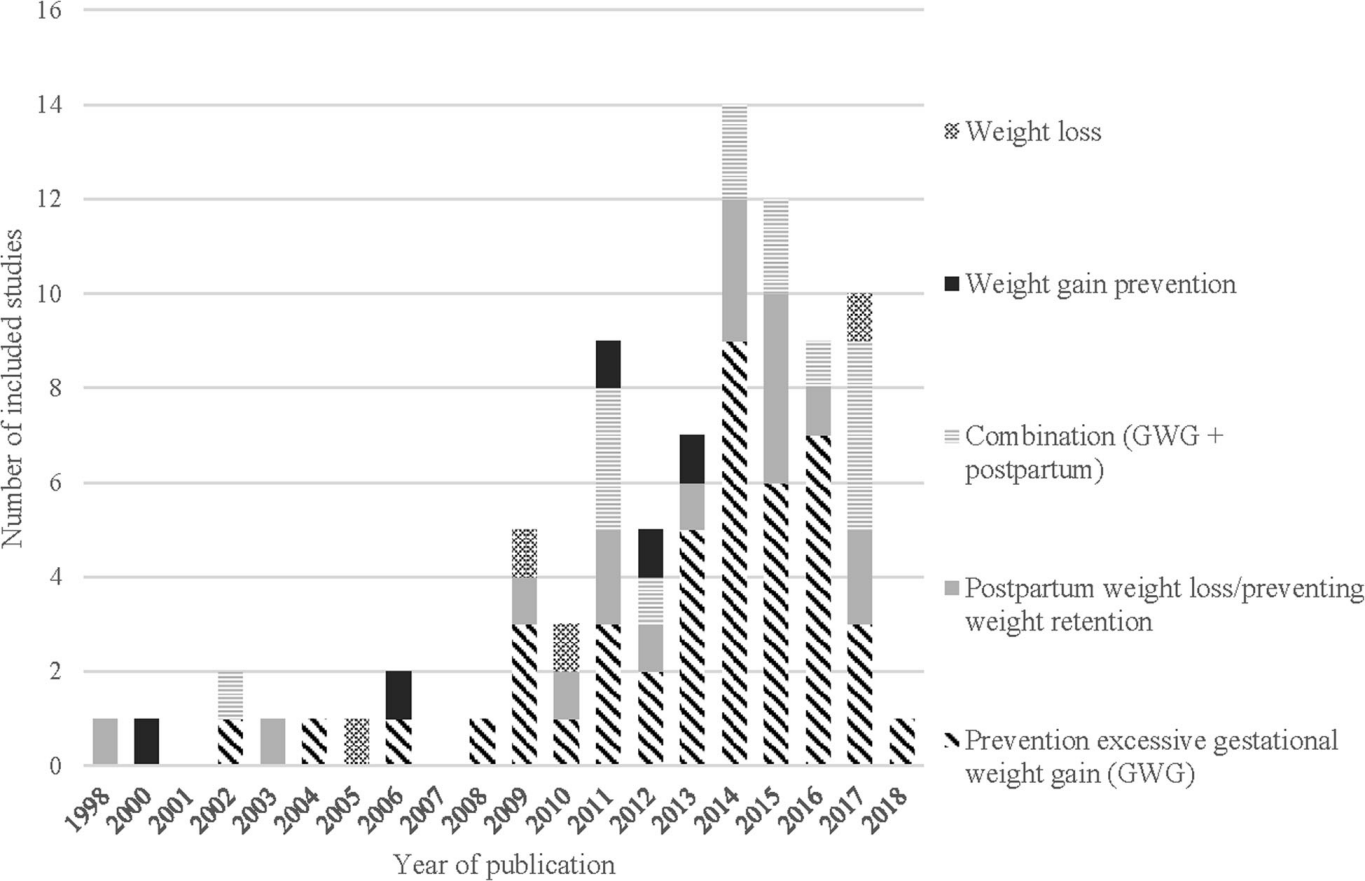
Number of included RCTs per year by weight focus

Figure 2 demonstrates the year of publication of the included RCTs, by weight focus. Studies were published from 1998 up until 2018, but with no studies published from 1998 to 2000, and only zero to two studies published per year from 2000 to 2008. From 2009 to 2017 the number of studies conducted per year ranged from three (2010) to 14 (2014). The intervention goal of included studies varied over time. While the number of studies focusing solely on postpartum weight loss/preventing weight retention has remained consistent focus over time, the number of weight gain prevention and weight loss studies unrelated to pregnancy decreased overtime, with more of a focus on prevention of excessive GWG, and the prevention of excessive GWG combined with postpartum weight loss/preventing postpartum weight retention.

Across the included RCTs there were 26,750 participants (Mean: 307.5). Many of the RCTs (59.8%, *n* = 52) recruited women of childbearing age as per our definition for inclusion (i.e. 18 to 44 years) or did not specifically define the age range for participant inclusion but implied they were ‘of childbearing’ age due to other inclusion criteria (i.e. pregnancy) (19.5%, *n* = 17). A significant number of RCTs (41.4%, *n* = 36) recruited only women who were affected by overweight or obesity, while many also did not report a BMI inclusion criteria (24.1%, *n* = 21).

Only five studies required women to be of a specific parity, with all five recruiting women during their first pregnancy. Twelve studies recruited participants from specific ethnicities, including African American (6.9%, *n* = 6) and Latina/Hispanic (5.7%, *n* = 5) women, and one study (1.1%) recruited only ‘white’ women. Ten RCTs (11.5%) had participant recruitment criteria related to the socio-economic status of the participant, with all specifically recruiting women of lower socio-economic status (e.g. lower income). Five studies recruited women with specific clinical conditions, including three studies (3.4%) that recruited women with gestational diabetes, one (1.1%) recruited women who were infertile, and one (1.1%) recruited women who were breastfeeding.

Table 2 describes the outcomes measured across the included RCTs. All studies measured weight related outcomes, with 78.2% (*n* = 62) having a weight-related primary outcome [27, 29–34, 36, 38, 40–44, 46, 47, 50, 51, 54, 56–59, 61–66, 68, 69, 71–80, 82–87, 90–103, 105, 106, 108–111]. Over half of the included RCTs (62.1%, *n* = 54) measured dietary and physical activity-related outcomes, with eight RCTs (9.2%) having a diet-related [45, 49, 51, 67, 71, 73, 75, 80] and five (5.7%) a physical activity related primary outcome [45, 49, 51, 73, 80]. At a minimum, all studies measured outcomes twice (i.e. baseline and follow-up), but the mean number of data collection points were 3.7 ± 2.0 (Range 2–14). There was a notable greater number of data collection points among studies focusing on GWG and preventing weight retention in the postpartum period (mean 5.1 ± 2.8).

**Table 2:** Summary of study outcomes by intervention goal and in total

		Total (*n* = 87)	General weight loss (*n* = 4)	Postpartum weight loss/preventing weight retention (*n* = 18)	General weight gain prevention (*n* = 5)	Excessive GWG prevention (*n* = 46)	Combined excessive GWG prevention and postpartum weight loss (*n* = 14)
Outcome measures- Number of data collection points	Mean, time points	3.7 ± 2.0	3.0 ± 0.7	2.6 ± 0.5	3.2 ± 0.8	3.8 ± 2.0	5.1 ± 2.8
	Median, time points	3	3	3	3	3	4
	Range, time points	2–14	2–4	2–3	2–4	2–10	2–14
	Unclear/not reported, n (%)	10 (11.5)	–	–	–	10 (21.7)	–
Weight	Primary outcome*, n (%)*	68 (78.2)	2 (50)	15 (83.3)	4 (80)	33 (71.7)	14 (100)
	Not reported if primary outcome, *n (%)*	3 (3.5)	–	3 (16.7)	–	–	–
	Not primary outcome, n (%)	16 (18.4)	2 (50)	–	1 (20)	13 (28.3)	–
Dietary behavior	An outcome*, n (%)*	54 (62.1)	2 (50)	16 (88.9)	2 (40)	26 (56.5)	8 (57.1)
	Primary outcome, *n (%)*	8 (9.2)	–	3 (16.7)	–	5 (10.9)	–
	Not reported if primary outcome, *n (%)*	5 (5.8)	1 (25)	2 (11.1)	–	2 (4.4)	–
	Not primary outcome, *n (%)*	41 (47.1)	1 (25)	11 (61.1)	2 (40)	19 (41.3)	8 (57.1)
Physical activity	An outcome*, n (%)*	54 (62.1)	2 (50)	14 (77.8)	3 (60)	26 (56.5)	9 (64.3)
	Primary outcome, *n (%)*	5 (5.8)	–	2 (11.1)	–	3 (6.5)	–
	Not reported if primary outcome, *n (%)*	5 (5.8)	1 (25)	2 (11.1)	–	2 (4.4)	–
	Not primary outcome, *n (%)*	44 (50.6)	1 (25)	10 (55.6)	3 (60)	21 (45.7)	9 (64.3)

Across the 87 included RCTs, there were 105 active intervention arms. Table 3 describes the characteristics of the 105 interventions overall and by weight focus. The majority (80.0%, *n* = 84) of the interventions focused on promoting both changes to dietary behavior and physical activity to achieve changes to weight-related outcomes. The interventions were most commonly delivered by those with expertise in nutrition, such as dietitians or nutritionists (42.9%, *n* = 45), or clinicians involved in the women’s care, such as midwives and/or general practitioners (GPs) (29.5%, *n* = 31). The setting for the interventions were commonly within the hospital or clinic (30.5%, *n* = 32), the participant’s home (28.6%, *n* = 30), or was not reported (27.6%, *n* = 29). Two-thirds (66.7%, *n* = 70) of interventions were delivered individually, 14 (13.3%) were group-based, and 23 (21.9%) used a combination of individual and group-based delivery.

**Table 3:** Summary of intervention characteristics by intervention goal and in total

		Total (*n* = 105)	General weight loss (*n* = 6 intervention arms)	Postpartum weight loss/preventing weight retention (*n* = 21 intervention arms)	General weight gain prevention (*n* = 6 intervention arms)	Excessive GWG prevention (*n* = 53 intervention arms)	Combined excessive GWG prevention and postpartum weight loss (*n* = 19 intervention arms)
Behavior change promoted	Dietary behavior only, *n (%)*	13 (12.4)	–	4 (19.1)	1 (16.7)	5 (9.4)	3 (15.8)
	Physical activity only, *n (%)*	5 (4.8)	–	2 (9.5)	–	2 (3.8)	1 (5.3)
	Combination, *n (%)*	85 (81)	6 (100)	15 (71.4)	5 (83.3)	44 (83.0)	16 (84.2)
Mode of delivery	Individual, *n (%)*	70 (66.7)	3 (50)	12 (57.1)	3 (50)	38 (71.7)	14 (73.7)
	Group, *n (%)*	14 (13.3)	2 (33.3)	2 (9.5)	2 (33.3)	6 (11.3)	2 (10.5)
	Combination, *n (%)*	23 (21.9)	1 (16.7)	7 (33.3)	1 (16.7)	10 (18.9)	4 (21.1)
Medium of delivery: Type	In-person, *n (%)*	85 (81.0)	5 (83.3)	17 (81.0)	3 (50)	43 (81.1)	17 (89.5)
	Telephone, *n (%)*	31 (29.5)	2 (33.3)	7 (33.3)	3 (50)	10 (18.9)	9 (47.4)
	Email, *n (%)*	7 (6.7)	2 (33.3)	1 (4.8)	2 (33.3)	1 (1.9)	1 (5.3)
	Web-based, *n (%)*	10 (9.5)	2 (33.3)	4 (19.1)	–	4 (7.6)	–
	Paper-based, *n (%)*	29 (27.6)	–	4 (19.1)	2 (33.3)	17 (32.1)	6 (31.6)
	Text message, *n (%)*	11 (10.5)	–	6 (28.6)	–	4 (7.6)	1 (5.3)
	Social media, *n (%)*	3 (2.9)	–	1 (4.8)	–	1 (1.9)	1 (5.3)
	Video, *n (%)*	7 (6.7)	1 (16.7)	1 (4.8)	–	5 (9.4)	–
	App, *n (%)*	1 (1.0)	–	1 (4.8)	–	–	–
Medium of delivery: Number used	One medium, *n (%)*	52 (49.5)	2 (33.3)	5 (23.8)	4 (66.7)	32 (60.4)	9 (47.4)
	Two media, *n (%)*	37 (35.2)	2 (33.3)	10 (47.6)	1 (16.7)	16 (30.2)	8 (42.1)
	Three media, *n (%)*	14 (13.3)	2 (33.3)	6 (28.6)	–	4 (7.6)	2 (10.5)
	Four media, *n (%)*	3 (2.9)	–	–	1 (16.7)	1 (1.9)	1 (5.3)
	Five media, *n (%)*	1 (1.0)	–	–	–	1 (1.9)	–
Profession of intervention deliverer	Dietetic professional (dietitian, nutritionist), *n (%)*	45 (42.9)	1 (16.7)	12 (57.1)	3 (50)	22 (41.5)	7 (36.8)
	Exercise professional (EP, physiotherapist, fitness trainers), *n (%)*	10 (9.5)	–	3 (14.3)	–	6 (11.3)	1 (5.3)
	Clinicians (nurses, midwifes, GP’s, obstetrician’s), *n (%)*	31 (29.5)	1 (16.7)	1 (4.8)	–	24 (45.3)	5 (26.3)
	Health coach/educators, *n (%)*	16 (15.2)	1 (16.7)	5 (23.8)	1 (16.7)	6 (11.3)	3 (15.8)
	Psychology professionals (counsellors), *n (%)*	6 (5.7)	2 (33.3)	2 (9.5)	–	2 (3.8)	–
	Research staff (research assistants, students, interventionists), n (%)	11 (10.5)	–	1 (4.8)	–	8 (15.1)	2 (10.5)
	Other, *n (%)*	2 (1.9)		–	–	1 (1.9)	1 (5.3)
	Not reported, *n (%)*	18 (17.1)	2 (33.3)	2 (9.5)	2 (33.3)	7 (13.2)	5 (26.3)
Setting	Home, *n (%)*	30 (28.6)	2 (33.3)	10 (47.6)	3 (50)	8 (15.1)	7 (36.8)
	Clinic/hospital, *n (%)*	32 (30.5)	1 (16.7)	1 (4.8)	–	24 (45.3)	6 (31.6)
	Community center, *n (%)*	10 (9.5)	–	3 (14.3)	2 (33.3)	4 (7.6)	1 (5.3)
	Research facility, *n (%)*	6 (5.7)	–	5 (23.8)	–	–	1 (5.3)
	Other, *n (%)*	8 (7.6)	–	3 (14.3)	1 (16.7)	2 (3.8)	2 (10.5)
	Not reported, *n (%)*	29 (27.6)	3 (50)	4 (19.1)	1 (16.7)	17 (32.1)	4 (21.1)

**Fig. 3:**
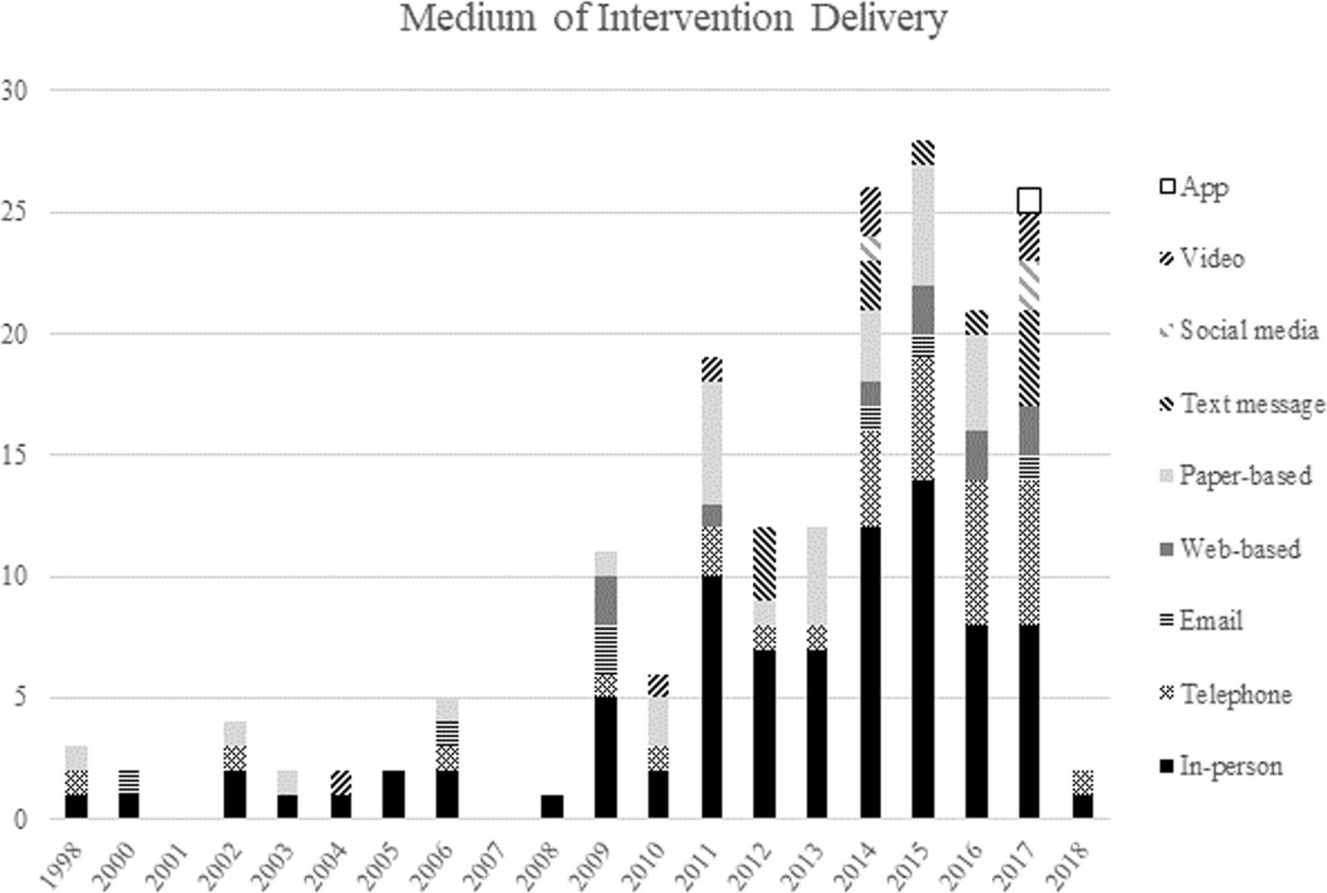
Medium of intervention delivery across included RCTs per year

Approximately half (49.5%, *n* = 52) of the interventions were delivered using one medium, and approximately one third (35.2%, *n* = 37) using two media. One study used five media to deliver the intervention. Most interventions (81.0%, *n* = 85) included components delivered in-person. A variety of other delivery mediums however were used across studies, including telephone (29.5%, *n* = 31), paper-based (27.6%, *n* = 29) (e.g. brochures), text message (10.5%, *n* = 11) and websites (9.5%, *n* = 10). Figure 3 shows the number of studies using different delivery modes by year of publication, demonstrating that the number of different delivery mediums utilized had increased over time.

### Systematic reviews

Three systematic reviews of RCTs were included in the scoping review, [15, 18, 113] which are described in detail in Additional file 1: Table S3. They included a total of 12 RCTs published from 1998 to 2011. Two of the reviews simply included studies with women of childbearing age, [18, 113] while the third included women of childbearing age ≥ 18 years of age [15]. Only one of the reviews specified that it only included participants affected by overweight or obesity [18]. None of the reviews had inclusion criteria related to participant’s ethnicity, clinical conditions or socio-economic status.

In terms of the reviews’ inclusion criteria for interventions, all reviews included studies with interventions that focused on modifying dietary intake, and two on modifying physical activity levels [15, 113]. Two of the reviews focused on prevention of excessive GWG, [15, 18] and one on prevention of excessive GWG and preventing weight retention in the postpartum period [113]. The reviews had no other specific inclusion criteria related to intervention types (e.g. mode of delivery, setting etc).

All three reviews required weight be reported as an outcome in the included studies, with one requiring it to be the primary outcome of the study [15]. There was no requirement in the three systematic reviews for diet or physical activity outcomes to be measured for the studies to be included in the review.

## Discussion

This is the first scoping review, to our knowledge, to comprehensively examine the extent and range of research undertaken to evaluate behavioral interventions that support women of childbearing age to prevent and treat overweight and obesity. The review identified 87 RCTs and three systematic reviews published in the last two decades. All of the systematic reviews addressed gestational weight gain, [15, 18, 113] and one also focused on postpartum weight retention [113]. There has been an increasing volume of research on supporting this age group of women through weight-related behavioral interventions over time, particularly in the last decade. The majority of studies were conducted in the United States and Australia although there was representation of studies from middle income (although not lower income) countries, supporting evidence from global data on the rising prevalence of women affected by overweight and obesity, that supporting women of childbearing age in weight management is an international issue [114].

Almost 90% of the RCTs aimed to support women to gain an appropriate amount of weight during pregnancy, and/or support weight loss or prevent weight retention following pregnancy. The surge in studies addressing excess gestational weight gain aligns with the 2009 publication of the revised United States Institute of Medicine (IOM) *Weight Gain during Pregnancy: Re-examining the Guidelines* [115]. National pregnancy guidelines of many countries, including Canada [116] and Australia, [117] follow the pregnancy weight gain recommended ranges established by the IOM (i.e. total weight gain during pregnancy of 12.5–18.0 kg for pre-pregnancy BMI < 18.5 kg/m^2^ [Underweight], 11.5–16.0 kg for pre-pregancy BMI of 18.5–24.9 kg/m^2^ [Normal weight], 7.0–11.5 kg for pre-pregnancy BMI of 25.0–29. 9 kg/m^2^ [Overweight], and 5.0–9.0 kg for pre-pregnancy BMI ≥30.0 kg/m^2^ [Obese]). There are many strengths to the approach of engaging women in weight management interventions during or following pregnancy. Pregnancy has been proposed as a time when effective behavioral interventions can impact the health of two generations, [118] potentially increasing the return for investment. There may be a heightened level of interest and motivation by women to address weight, healthy eating and physical activity behaviors to improve pregnancy and health outcomes for themselves and their child [118, 119]. It may also be easier to reach women through existing routine antenatal and postnatal health care services when they come in to regular contact with a range of health care providers including doctors, midwives, nurses, dietitians, pharmacists, and reproductive health specialists [120].

However, there are limitations to heavily relying on pregnancy and postpartum interventions. Pregnant women are typically recruited to participate in gestational weight gain behavioral interventions midway through their second trimester [121]. This limits the potential impact of the intervention to support weight management since women who conceive with an obese, overweight or healthy weight BMI on average surpass their recommended weight gain by 18, 20 and 30 weeks of pregnancy, respectively [122]. First trimester weight gain in excess of 0.5–2 kg is also predictive of excess gestational weight gain during pregnancy [123]. Behavioral interventions that engage and support women to gain an appropriate amount of weight from earlier in pregnancy are needed [124]. However, this brings further clinical practice implementation challenges when up to 30–40% of woman do not begin receiving antenatal care until their second trimester [125, 126]. Given that pregnancy behavioral interventions have resulted in only modest reductions in gestational weight gain (of about 0.7 kg), and few have improved maternal and child health outcomes, there are compelling calls for future intervention research to also focus on the preconception period [127].

There is growing evidence on the role of obesity in preconception as a major determinant of offspring health in childhood and later adult life through the developmental origins of health and disease hypothesis [128]. In pregnant women, a higher pre-pregnancy BMI has been consistently identified as a strong predictor of pregnancy complications [129] and adverse offspring non-communicable health trajectories [130–132]. For example, when compared to a healthy pre-pregnancy BMI (18.5–24.9 kg/m^2^) a pre-pregnancy BMI of ≥40 kg/m^2^ has been shown to be associated with increased risk of gestational diabetes (Odds Ratio: 11.01 95% confidence interval 10.25–11.82), preeclampsia (OR: 4.44, 95%CI:4.17–4.72) and pre-term birth (OR: 2.91 95% CI: 2.21–3.81) [129]. The scoping review identified only one behavioral intervention *directly* supporting women in the preconception period who were planning a pregnancy. While the opportunity to support women at the individual level who are actively planning a pregnancy is important, it is also limited by low preconception care engagement (60–80% of women don’t receive preconception care [133–135]) and unplanned pregnancies, with global estimates that 44% of all pregnancies are unintended [136].

By only addressing weight management associated with a pregnancy event, an opportunity is missed to support weight management in the young female population, regardless of their intention or ability to have children. Only nine intervention studies that were unrelated to pregnancy were identified through the scoping review demonstrating a substantial research gap. Women tend to gain 0.5–1 kg each year from early adulthood until middle-age, [137, 138] with all young women, not only those who bear children, at risk of unhealthy weight gain [7]. An 18 year follow-up of 92,837 women from early to mid-adulthood in the Nurses’ Health Study found that women who gained between 2.5–10 kg had an increased incidence of type II diabetes, cardiovascular disease, obesity-related cancer, and mortality [8]. Effective behavioral interventions to support the entire young adult female population to improve health behaviors and weight management will improve chronic disease health trajectories more broadly, with added reproductive and intergenerational health benefits for women whose future pregnancies are both planned and unplanned.

Few weight management behavioral studies, with weight as the primary focus, have been conducted with women with clinical conditions. Engaging young women with existing or previous clinical conditions, such as type II diabetes, polycystic ovary syndrome, pre-eclampsia, and women who are experiencing obesity-related infertility, may help to reach and support young women at higher risk of obesity. More than half of the studies in this scoping review only recruited women who were affected by overweight and/or obesity. Weight gain prevention research to support young women to maintain their weight is needed if the rising obesity epidemic is to be halted. More research is also needed with populations under-represented in this review, such as women from lower socioeconomic groups and culturally diverse populations who may experience other barriers to weight management and have different patterns of health service engagement. Researchers should draw on existing behavioral modification research with lower socioeconomic groups and culturally diverse population groups to inform intervention design. Developing a better understanding of effective socially and culturally appropriate behavioral support, and clinician barriers to providing best practice care (e.g. clinician BMI and personal weight satisfaction may influence their confidence in providing best practice care [139]), could inform the delivery of population and primary health care initiatives for weight management.

The interventions included a variety of delivery modes and mediums, with individual and in-person delivery most commonly used. While the number of different delivery mediums used has increased over-time, no consistent or proven delivery medium has emerged [140]. Half of the interventions used more than one type of medium, such as in-person, telephone and text messages. Interventions delivered in the postpartum period tended to use more than one medium, and a greater variety of mediums. The use of multiple communication avenues may highlight efforts to overcome the difficulty of reaching new mothers who face complex barriers to participation, including a lack of time and need for childcare [141, 142]. Identifying whether one or more of the delivery modes and mediums are more effective in helping young women to manage their weight could help to inform the delivery of future interventions. Assessment of the intervention feasibility and acceptability by those delivering, and women receiving, weight management care should also be undertaken. Interventions were commonly provided by dietitians or nutritionists, or health care providers such as midwives and GPs. However, half of the studies did not deliver care through a dietetic or exercise professional, despite the professions’ expertise in nutrition [143, 144] and physical activity [145] for weight management. Systematic review evidence has shown that weight management interventions delivered by health care providers, [146] including dietitians, [147] are more effective than those delivered by non-health care providers.

This review has several strengths. It is the first scoping review to comprehensively examine the extent and range of research undertaken to evaluate behavioral interventions that support women of childbearing age to prevent and treat overweight and obesity. The review employed a comprehensive search strategy across numerous databases, and summarized the evidence from systematic reviews and RCTs, the two highest levels of evidence [26]. However, by limiting to systematic reviews and RCTs, evaluations of relevant interventions using other experimental study designs (e.g. pre-post studies, non-randomized control trials) were excluded from the review. In addition, the review only considered the extent and range of studies, and did not explore the efficacy of the interventions. Another main limitation of the scoping review was the challenge of combining data of studies that spanned different life periods (e.g. preconception, pregnancy, postpartum, non-pregnancy related) to accurately describe intervention duration and the timing of measurement outcomes, as they were variable within studies (e.g. due to timing of intervention delivery approximately based on weeks’ gestation in pregnancy) and across studies (e.g. due to differences in health service delivery, and the timing of when women are seen during and after pregnancy). Further, while 92 and 85% of studies reported interventions that targeted diet and/or physical activity behaviour change, respectively, only 62% of studies measured diet and 62% measured physical activity as outcomes. There is a need for studies to include valid and reliable diet and physical activity outcomes, particularly to investigate changes in diet and physical activity as a mediator of weight change. Finally, the scoping review included studies published up until 31st January 2018, therefore it is possible that additional studies meeting the inclusion criteria have been published since that time.

## Conclusions

There is a substantial body of research from the two highest levels of evidence on behavioral interventions that support women of childbearing age to prevent and treat overweight and obesity, particularly from research published within the last decade. The majority has focused on weight management during or after a pregnancy event, demonstrating a research gap to support weight management in the young adult female population in preconception and unrelated to pregnancy to improve their own chronic disease health trajectories, with reproductive and intergenerational health benefits for future planned and unplanned pregnancies. Future research to examine delivery modes and mediums, optimal intervention duration and intensity, involvement of health care providers, and involvement of under-represented populations should be considered, both to understand effective behavioral interventions, and to ensure that interventions are scalable and can be implemented within policy and practice, such as through population and primary health care.

## Supplementary information

**Additional file 1:** **Table S1.** Full electronic search for one database (MEDLINE). **Table S2.** Summary of included Randomised Control Trials. **Table S3.** Summary of Included Systematic Reviews.

## Data Availability

All data generated or analyzed during this study are included in this published article [and its supplementary information files]. The databases utilized in the search strategy were accessed via institutional licenses of the University of Newcastle, and therefore public access to the databases is closed.

## Abbreviations

BMI: Body mass index
GWG: Gestational weight gain
IOM: Institute of Medicine
RCT: Randomized controlled trial
