# Supplementary material for: Supporting women of childbearing age in the prevention and treatment of overweight and obesity: a scoping review of randomized control trials of behavioral interventions
Source: BMC Womens Health. 2020 Jan 23;20:14. doi: 10.1186/s12905-020-0882-3 (PMC6979060; doi:10.1186/s12905-020-0882-3)
Supplement: Supplementary file 1 — Additional file 1: Table S1. Full electronic search for one database (MEDLINE). Table S2. Summary of included Randomised Control Trials. Table S3. Summary of Included Systematic Reviews. [file 12905_2020_882_MOESM1_ESM.pdf]

Table S1. Full electronic search for one database (MEDLINE)

#	Searches	Results
1	young wom?n.mp.	26887
2	middle age* wom?n.mp.	4371
3	premenopause/ or pre-menopausal.mp.	8381
4	childbearing.mp.	11514
5	child bearing.mp.	2522
6	pregnan*.mp.	918461
7	(pre-conception or preconception).mp.	4261
8	(postpartum or postnatal).mp. or Postpartum Period/	151583
9	maternal.mp.	270982
10	1 or 2 or 3 or 4 or 5 or 6 or 7 or 8 or 9	1118037
11	(weight gain or weight loss or weight maintenance or weight reduction or weight control or weight management or obesity prevention).mp.	155916
12	((behavi* or lifestyle or diet* or activit* or exercise or sedentary) adj5 intervention*).mp.	53958
13	11 or 12	203190
14	randomized controlled trial.mp. or Randomized Controlled Trial/	471806
15	clinical trial.mp. or clinical trial/	651558
16	random*.tw.	953114
17	trial.tw.	489225
18	systematic review*.mp.	114555
19	meta-analysis.mp.	136587
20	14 or 15 or 16 or 17 or 18 or 19	1679945
21	10 and 13 and 20	3542
22	limit 21 to (english language and humans)	2509

Table S2. Summary of included Randomised Control Trials

Author	Year	Country of origin	Sample size	Age	BMI	Pregnancy related	Ethnicity	SES	Clinical Condition	No. study arms	Number of intervention arms	Intervention focus *	Behaviour change	Mode of delivery	Medium of delivery	Profession intervention deliverer	Setting	Weight related primary outcome	Diet outcome	Physical activity outcome
Daley [1]	2015	UK	76	≥18	18-29.9	Antenatal	NC	NC	NC	2	1	A	Diet + PA	Individual	In-person	Community midwife	Maternity centre	No	No	Yes
Di Carlo [2]	2014	Italy	154	NC	20-40	Antenatal	NC	NC	NC	2	1	A	Diet	Individual	In-person	Dietitian	Antenatal clinic	Yes	No	No
Dodd, Grivell [3-8]	2014	Australia	2212	NC	≥25	Antenatal	NC	NC	NC	2	1	A	Diet + PA	Combination	Telephone, in-person	Dietitian, research assistants	NR	No	Yes	Yes
Eiben [9]	2006	Sweden	40	18-28	≥18.5	No	NC	NC	NC	2	1	C	Diet + PA	Combination	In-person, telephone, email, paper-based	Dietitian	Home	Yes	Yes	Yes
Farajzadegan [10]	2013	Iran	160	NC	NC	Antenatal	NC	NC	NC	2	1	A	Diet + PA	Individual	In-person, paper-based	Midwife	Public and private obstetric offices	Yes	No	No
Ferrara [11]	2016	USA	2280	≥18	NC	Antenatal	NC	NC	GDM	2	1	A and B	Diet + PA	Individual	Paper-based, telephone	Dietitian	KPNC (integrated health care delivery system)	Yes	Yes	Yes
Foley [12]	2012	USA	194	25-44	25-34.9	No	Black women	NC	NC	2	1	C	Diet + PA	Individual	Paper-based, telephone	Dietitian	Community health centre	Yes	No	Yes
Gesell [13]	2015	USA	135	≥16	NC	Antenatal	Predominately Latina women	NC	NC	2	1	A	Diet + PA	Group	In-person	Health care provider	Community recreation center	No	No	No
Gilmore [14]	2017	USA	40	≥18	25-40	Postnatal	NC	NC	NC	2	1	B	Diet + PA	Individual	App	Dietitian	Home	Yes	No	No
Graham [15]	2017	USA	1335	18-35	18.5-34.9	Antenatal	NC	NC	NC	2	1	A	Diet + PA	Individual	Web-based, email	NR	Home	Yes	No	No
Guelinckx [16]	2010	Belgium	195	NC	>29.0	Antenatal	White women	NC	NC	3	2	A	Diet + PA	Combination	In-person, paper-based	Nutritionist	NR	Yes	Yes	Yes
													Diet + PA	Individual	Paper-based	NR	NR			
Haakstad [17]	2011	Norway	105	NC	NC	Antenatal	NC	NC	NC	2	1	A and B	Diet + PA	Group	In-person	Aerobic instructors	NR	Yes	No	Yes
Haby [18]	2015	Sweden	100	NC	30-39.9	Antenatal	NC	NC	NC	2	1	A	Diet + PA	Combination	In-person	Midwife, dietitian	Midwifery practice	Yes	No	No
Haire-Joshu [19]	2015	USA	1325	Adolescents	NC	Postnatal	NC	NC	NC	2	1	B	Diet + PA	Combination	In-person, paper-based, web-based	Parent educator	Schools, homes	Yes	Yes	Yes
Harden [20]	2014	USA	16	21-35	>30.0	Antenatal	NC	Low income	NC	2	1	A	Diet + PA	Group	In-person	Graduate student with expertise in group dynamics and lifestyle interventions during pregnancy	Clinic	Yes	No	No
Harden [20]	2014	USA	51	18-45	≥18.5	Antenatal	NC	NC	NC	2	2	A	Diet + PA	Group	In-person	Graduate student with expertise in group dynamics and lifestyle interventions during pregnancy	Fitness facility	Yes	No	No
													Diet + PA	Group	In-person, paper-based	Graduate student with expertise in	Fitness facility			

																group dynamics and lifestyle interventions during pregnancy				
Harrison [21-23]	2013	Australia	228	NC	≥25 or ≥23 if high-risk ethnicity	Antenatal	NC	NC	NC	2	1	A	Diet + PA	Individual	In-person	Exercise physiologist	Antenatal clinic	Yes	No	Yes
Hawkins [24]	2015	USA	68	18-40	≥25 pre-pregnancy BMI	Antenatal	Hispanic	NC	NC	2	1	A	Diet + PA	Individual	In-person, telephone, paper-based	Health educator	NR	No	Yes (P)	Yes (P)
Herring [25, 26]	2017	USA	66	≥18	25-45 first trimester BMI	Antenatal	African American	Low income - Medicaid recipient (income proxy)	NC	2	1	A and B	Diet + PA	Individual	Text messages, telephone, Facebook, in-person	Bachelor-level health coach	Home	Yes	No	No
Herring [27]	2014	USA	18	≥18	≥25 first trimester BMI	Postnatal	NC	NC	NC	2	1	B	Diet + PA	Individual	Text message, telephone, social media	Bachelor-level health coach	Home	Yes	Yes	Yes
Huang [28]	2011	Taiwan	240	≥18	NC	Antenatal	NC	NC	NC	3	2	A and B	Diet + PA	Individual	In-person, paper-based	Nurse	Clinic	Yes	Yes	Yes
													Diet + PA	Individual	In-person, paper-based	Nurse	Clinic			
Hui [29]	2012	Canada	224	NC	NC	Antenatal	NC	NC	NC	2	1	A	Diet + PA	Combination	In-person	Fitness trainers, dietitian	Community centres	Yes	Yes	Yes
Hui [30]	2014	Canada	113	NC	NC	Antenatal	NC	NC	NC	2	1	A	Diet + PA	Combination	In-person, video	Dietitian	NR	Yes	Yes	Yes
Huseinovic [31]	2016	Sweden	110	NC	≥27	Postnatal	NC	NC	NC	2	1	B	Diet	Individual	In-person, text message, telephone	Dietitian	Primary health care clinic	Yes	Yes (P)	Yes (P)
Jackson [32]	2011	USA	321	≥18	NC	Antenatal	NC	NC	NC	2	1	A	Diet + PA	Individual	Computer program/video, paper-based	Clinician	Clinic	No	Yes (P)	Yes (P)
Jeffries [33]	2009	Australia	286	18-45	NC	Antenatal	NC	NC	NC	2	1	A	Weighing	Individual	Paper-based (personalised weight-measurement card)	NR	Home, clinic	Yes	No	No
Jing [34]	2015	China	262	≥18	NC	Antenatal	NC	NC	NC	2	1	A	Diet + PA	Individual	In-person, paper-based and telephone	Graduate student	NR	Yes	Yes (P)	Yes (P)
John [35]	2014	UK	570	≥18	≥30	Antenatal	NC	NC	NC	2	2	A and B	Diet + PA	Combination	In-person, telephone	Midwife and a 'Slimming World' consultant	Antenatal clinic	Yes	Yes	Yes
													Diet + PA	Individual	Paper-based	NR	NR			
Keller [36]	2014	USA	139	18-40	25-35	Postnatal	Latina	NC	NC	1	1	B	Diet	Group	In-person	Promotoras	Community	NR	Yes	Yes
Kinnunen [37]	2012	Finland	442	≥18	≥ 25 (or other GDM risk factors)	Antenatal	NC	NC	NC	2	1	A	Diet + PA	Individual	In-person	Nurse	Maternity clinic	No	No	No
Klem [38]	2000	USA	102	25-34	21-25	No	NC	NC	NC	3	2	C	Diet + PA	Group	In-person	NR	NR	No	No	No
													Diet + PA	Individual	Email	NR	Home			
Koivusalo [39]	2016	Finland	293	≥18	≥30 and/or with a	Antenatal	NC	NC	NC	2	1	A	Diet + PA	Combination	In-person	Nurse, dietitian	NR	No	Yes	Yes

					history of GDM															
Lim [40]	2009	Australia	203	17-37	25.1-44	No	NC	NC	NC	2	2	D	Diet + PA	Individual	In-person, web-based, email	NR	Clinical setting	Yes	Yes	Yes
													Diet + PA	Individual	In-person, web-based, email	NR	Home			
Martin [41]	2015	Australia	36	>18	25-35	Antenatal	NC	NC	NC	3	2	A and B	Diet	Individual	In-person, telephone	Dietitian, lactation consultant	Hospital	Yes	No	No
													Diet	Individual	In-person	Dietitian	Hospital			
McCarthy [42]	2016	Australia	382	≥18	≥25	Antenatal	NC	NC	NC	2	1	A	Diet	Individual	In-person	Research midwife	Antenatal clinic in a hospital	No	No	No
Mutsaerts, van Dammen [43-45]	2017	Netherlands	577	18-39	29-40	No	NC	NC	Infertile	2	1	D	Diet + PA	Individual	In-person, telephone	Nurse, dietitian	University medical centres and general hospitals	No	No	No
Nicklas [46]	2014	USA	75	18-45	24.1-50, ≥22.1 for Asian participants.	Postnatal	NC	NC	GDM	2	1	B	Diet + PA	Individual	Web-based, telephone, email	Dietitian, physician	Home	Yes	Yes	Yes
Østbye [47]	2009	USA	450	≥18	≥25 pre-pregnancy BMI	Postnatal	NC	NC	NC	2	1	B	Diet + PA	Combination	In-person, telephone	Counselor	NR	Yes	Yes	Yes
Peccei [48]	2017	USA	300	18-49	25-40	Antenatal	NC	NC	NC	2	1	A and B	Diet + PA	Individual	In-person, telephone	Dietitian	Community health setting	Yes	No	No
Petrella [49]	2014	Italy	61	>18	>25	Antenatal	NC	NC	NC	2	1	A	Diet + PA	Individual	In-person	Gynaecologist, dietitian	Antenatal clinic	Yes	Yes	Yes
Phelan [50, 51]	2015	USA	408	18-40	≥25 or have a BMI between 22 and 24.9 and exceed pre-pregnancy weight by at least 4.5 kg	Postnatal	NC	Low income	NC	2	1	B	Diet + PA	Combination	In-person, web-based, text message	NR	Home	Yes	Yes	Yes
Phelan [52, 53]	2011	USA	401	>18	19.8-40	Antenatal	NC	NC	NC	2	1	A and B	Diet + PA	Individual	In-person, mail (paper-based), telephone	Dietitian, interventionist	Home	Yes	Yes	Yes
Pollak [54]	2014	USA	35	≥18	25-40	Antenatal	NC	NC	NC	2	1	A	Diet + PA	Individual	Text message	NR	Home	Yes	Yes	Yes
Polley [55]	2002	USA	120	≥18	≥19.8	Antenatal	NC	Low income	NC	2	1	A	Diet + PA	Individual	In-person, telephone, mail (paper-based)	Master's and doctoral level staff with training in nutrition or clinical psychology	Clinic	Yes	Yes	Yes
Quinlivan [56]	2011	Australia	132	NC	≥25	Antenatal	NC	NC	NC	2	1	A	Diet	Individual	In-person	Midwife, obstetrician, general practitioner, food technologist	Antenatal clinic	No	Yes	No
Rauh [57, 58]	2013	Germany	250	>18	≥18.5	Antenatal	NC	NC	NC	2	1	A	Diet + PA	Individual	In-person	Trained researchers	NR	Yes	Yes	Yes

Rauh [59]	2014	Germany	2500	18-43	18.5-40	Antenatal	NC	NC	NC	2	1	A	Diet + PA	Individual	In-person	Midwife, gynaecologist, medical staff	Antenatal clinic	Yes	Yes	Yes
Renault [60]	2017	Denmark	425	≥18	≥30	Antenatal	NC	NC	NC	3	2	A	Diet + PA	Individual	In-person, telephone, text message	Dietitian	Outpatient Clinic	Yes	Yes	Yes
													PA	Individual	In-person, text message	Dietitian	Home			
Ronnberg [61, 62]	2015	Sweden	445	≥18	≥19	Antenatal	NC	NC	NC	2	1	A	PA	Individual	In-person	Midwife	Antenatal clinic	Yes	No	No
Sagedal, Sanda [63-65]	2017	Norway	606	≥18	≥19	Antenatal	NC	NC	NC	2	1	A and B	Diet + PA	Combination	In-person, telephone	Clinical dietitian or graduate students in public health, physical therapist or students in sports science	Gym, home	Yes	No	Yes
Skouteris [66]	2016	Australia	261	≥18	NC	Antenatal	NC	NC	NC	2	2	A	Diet + PA	Combination	In-person, telephone	Health coach	NR	Yes	No	No
													Diet + PA	Group	In-person	Health coach	NR			
Smith [67]	2016	USA	51	18-45	≥18.5	Antenatal	NC	NC	NC	2	1	A	Diet + PA	Individual	Web-based	NR	Home	Yes	Yes	Yes
Stendell-Hollis [68]	2013	USA	138	18-40	NC	Postnatal	NC	NC	breastfeeding	2	2	B	Diet	Individual	In-person	Dietitian	Study clinic	NR	Yes	No
													Diet	Individual	In-person	Dietitian	Study clinic			
Szmeja [69]	2014	Australia	1105	Child bearing'	≥25	Antenatal	NC	NC	NC	2	2	A	Diet + PA	Individual	Video	Research dietitian	NR	No	Yes (P)	Yes
													Diet + PA	Individual	Paper-based	Research dietitian	NR			
Tawfik [70]	2017	Egypt	235	18-45	NC	Antenatal	NC	NC	GDM	2	1	A and B	Diet + PA	Individual	In-person	Physician	NR	Yes	No	No
Thomson [71, 72]	2014	USA	82	≥18	NC	Antenatal	NC	NC	NC	2	2	A and B	Diet + PA	Individual	In-person	Community-based, trained Parent Educators	Home	Yes	Yes	Yes
													Diet + PA	Combination	In-person	Community-based, trained Parent Educators	Home			
Thornton [73]	2009	USA	100	18-49	<40	Antenatal	NC	NC	NC	2	1	A	Diet + PA	Individual	In-person	Dietitian	NR	Yes	No	No
Vesco [74-76]	2012	USA	114	≥18	≥30	Antenatal	NC	NC	NC	2	2	A and B	Diet + PA	Combination	In-person	Dietitian	Centre for Health Research Clinic	Yes	Yes	Yes
													Diet + PA	Individual	In-person	NR	NR			
Vinter [77-79]	2011	Denmark	360	18-40	30-45	Antenatal	NC	NC	NC	2	1	A	Diet + PA	Combination	In-person	Dietitian, physiotherapist, physician, midwife	Hospital	Yes	No	Yes
Wilkinson [80]	2015	Australia	71	> 18 years or younger than 18 with consent of parent or	≥25	Postnatal	NC	NC	NC	2	1	B	Diet + PA	Individual	In-person, paper-based	Dietitian	NR	NR	Yes	Yes

				guardian																
Willcox [81]	2017	Australia	100	≥18	>25	Antenatal	NC	NC	NC	2	1	A	Diet + PA	Individual	Text message, video, social media, in-person, web-based	Obstetrician, dietitian, physiotherapist	Home	No	Yes	Yes
Wolff [82]	2008	Denmark	66	18-45	≥30	Antenatal	NC	NC	NC	2	1	A	Diet	Individual	In-person	Dietitian	NR	Yes	Yes (P)	No
Abdel-Aziz [83]	2018	Egypt	200	20-30	NC	Antenatal	NC	NC	NC	2	1	A	Diet + PA	Individual	In-person, telephone	Nutrition counselor	Antenatal care clinic in hospital	Yes	Yes	Yes
Adamo [84]	2013	Canada	60	≥18	>18.5	Antenatal	NC	NC	NC	2	1	A	Diet + PA	Combination	Paper-based, in-person	Exercise physiologist, dietitian, nutritionist	NR	No	Yes	Yes
Althuizen [85, 86]	2006	Netherlands	246	Childbearing'	NC	Antenatal	NC	NC	NC	2	1	A	Diet + PA	Individual	In-person	Counselor	Midwifery practice	Yes	Yes	Yes
Ames [87]	2005	USA	67	18-30	28-40	No	NC	NC	NC	2	2	D	Diet + PA	Group	In-person	Psychology graduates	NR	No	No	No
													Diet + PA	Group	In-person	Psychology graduates	NR			
Asbee [88]	2009	USA	144	18-49	</=40	Antenatal	NC	NC	NC	2	1	A	Diet + PA	Individual	In-person	Dietitian, physician, nurse practitioner	Obstetrics Clinic	Yes	No	No
Asci [89]	2016	Turkey	102	>18	NC	Antenatal	NC	NC	NC	2	1	A	Diet + PA	Individual	In-person	Researcher	Family health center	Yes	Yes	Yes
Bechtel-Blackwell [90]	2002	USA	46	13-18	NC	Antenatal	African American	NC	NC	2	1	A and B	Diet	Group	In-person	NR	Adolescent prenatal clinic	Yes	Yes	No
Bennett [91]	2013	USA	194	25-44	25-34.9	No	Black women	Socioeconomically disadvantaged	NC	2	1	C	Diet	Individual	Telephone	Dietitian	Home	Yes	No	No
Berry [92]	2011	USA	56	18-43	≥25	No	Hispanic	NC	NC	2	1	C	Diet + PA	Group	In-person	Community health educator	Local church and community centre	Yes	Yes	Yes
Berry [93]	2015	USA	60	≥18	>25	Postnatal	NC	NC	NC	2	1	B	Diet + PA	Group	In-person	Health education interventionist	Community-based health clinic	Yes	Yes	Yes
Bertz, Brekke, Huseinovic [94-97]	2012	Sweden	68	Childbearing'	25-35	Postnatal	NC	NC	NC	4	3	B	Diet	Individual	In-person, text message	Dietitian	Research clinic and home	Yes	Yes (P)	Yes (P)
													PA	Individual	In-person, text message	Physical therapist	Home			
													Diet + PA	Individual	In-person, text message	Dietitian and physical therapist	Research clinic and home			
Bogaerts [98]	2013	Belgium	205	Childbearing'	≥29	Antenatal	NC	NC	NC	3	2	A	Diet + PA	Individual	Paper-based	NR	Home	Yes	No	No
													Diet + PA	Group	In-person, paper-based	Midwife	NR			
Brownfoot [99]	2016	Australia	782	18-45	NC	Antenatal	NC	NC	NC	2	1	A	Non-specific	Individual	In-person	Clinician	Antenatal clinic	Yes	No	No
Chang [100]	2010	USA	129	18-34	25.0-39.9	NC	African American or non-Hispanics	low-income	NC	2	1	D	Diet + PA	Combination	Video, telephone	WIC educator	Home	Yes	Yes	Yes

Chang [101, 102]	2017	USA	612	18-39	25.0-39.9	Postnatal	African American or non-Hispanics	low-income	NC	2	1	B	Diet + PA	Combination	Video, telephone	WIC educator	Home	Yes	No	No
Chasan-Taber [103]	2015	USA	300	18-45	≥25	Antenatal	Hispanic	NC	NC	2	1	A and B	Diet + PA	Individual	In-person, paper-based and telephone	NR	Home	Yes	Yes	Yes
Clements [104]	2016	Australia	710	≥18	≥18.5	Antenatal	NC	NC	NC	2	2	A	Diet + PA	Individual	Telephone, paper-based	Health coaches	Antenatal clinic, hospital	Yes	Yes	No
													Diet + PA	Individual	Telephone, paper-based	Health coaches	Antenatal clinic, hospital			
Colleran [105]	2012	USA	31	23-37	25-30	Postnatal	NC	NC	NC	2	1	B	Diet + PA	Individual	In-person, online	Dietitian, research assistants	Home	Yes	Yes (P)	No
Craigie [106]	2011	UK	52	16-50	>25	Postnatal	NC	Living in areas of moderate to high deprivation	NC	2	1	B	Diet + PA	Individual	In-person, telephone	Trained lifestyle counsellor	A location most convenient for the participant	Yes	Yes	Yes
Hui [107]	2006	Canada	52	Child bearing'	NC	Antenatal	NC	Socioeconomically deprived living in an urban core.	NC	2	1	A	Diet + PA	Combination	In-person, video	Professional trainers (exercise), student assistants, dietitians	Community centre	No	Yes	Yes
Krummel [108]	2010	USA	151	≥18	Not underweight	Postnatal	NC	low-income	NC	2	1	B	Diet + PA	Combination	In-person, newsletters	Nutritionists facilitated the group discussions, dietitian conducted the counselling session	WIC offices, church basement and YMCAs	Yes	Yes	Yes
Leermakers [109]	1998	USA	90	≥18	≥22	Postnatal	NC	NC	NC	2	1	B	Diet + PA	Combination	In-person, paper-based via mail, telephone	NR	NR	Yes	Yes	Yes
O'Toole [110]	2003	USA	40	Child bearing'	25-29.9 prior to pregnancy	Postnatal	NC	NC	NC	2	1	B	Diet + PA	Combination	In-person, paper-based	Dietitian, exercise physiologist	University exercise research lab	Yes	Yes	Yes
Nagle [111]	2011	Australia	214	Child bearing'	≥30	Antenatal	NC	NC	NC	2	1	A	Diet	Individual	In-person, paper-based	Midwife	Hospital	Yes	No	No
LeBlanc [112]	2016	USA	150	18-40	≥ 27	No (planning in next 2 years)	NC	NC	NC	2	1	A	Diet + PA	Individual	In-person, paper-based, online, telephone	Health coach	NR	Yes	Yes	Yes

* Intervention focus. A = excessive GWG prevention; B = Postpartum weight loss/preventing weight retention; C = General weight gain prevention; D = General weight loss

Table S3. Summary of Included Systematic reviews inclusion criteria

Author	Year	Number of RCTs included	Age	BMI	If yes, what?	Ethnicity	SES	Clinical Condition	Intervention focus	Behaviour change focus	Mode of delivery as inclusion criteria? (y/n)	Medium of delivery as inclusion criteria (y/n)	Profession intervention deliverer as inclusion criteria? (y/n)	Setting as inclusion criteria? (y/n)	Weight-related outcome	Diet outcome as inclusion criteria? (y/n)	Physical activity outcome as inclusion criteria? (y/n)
Brown [113]	2012	5	≥18	NA	Antenatal	No	No	No	Excessive GWG prevention	Diet + PA	No	No	No	No	Yes (P)	No	No
Kuhlmann [114]	2008	3	NA	NA	Prenatal, antenatal and postnatal	No	No	No	Excessive GWG prevention and postpartum weight loss	Diet + PA	No	No	No	No	Yes	No	No
Quinlivan [115]	2011	4	NA	≥25	Antenatal	No	No	No	Excessive GWG prevention	PA	No	No	No	No	Yes	No	No

References

1. Daley AJ, Jolly K, Jebb SA, Lewis AL, Clifford S, Roalfe AK, Kenyon S, Aveyard P: **Feasibility and acceptability of regular weighing, setting weight gain limits and providing feedback by community midwives to prevent excess weight gain during pregnancy: Randomised controlled trial and qualitative study**. *BMC Obesity* 2015, **2 (1)**(35).
2. Di Carlo C, Iannotti G, Sparice S, Chiacchio MP, Greco E, Tommaselli GA, Nappi C: **The role of a personalized dietary intervention in managing gestational weight gain: a prospective, controlled study in a low-risk antenatal population**. *Arch Gynecol Obstet* 2014, **289**(4):765-770.
3. Dodd JM, Cramp C, Sui Z, Yelland LN, Deussen AR, Grivell RM, Moran LJ, Crowther CA, Turnbull D, McPhee AJ *et al*: **The effects of antenatal dietary and lifestyle advice for women who are overweight or obese on maternal diet and physical activity: The LIMIT randomised trial**. *BMC Medicine* 2014, **12 (1)**(161).
4. Grivell RM, Yelland LN, Deussen A, Crowther CA, Dodd JM: **Antenatal dietary and lifestyle advice for women who are overweight or obese and the effect on fetal growth and adiposity: the LIMIT randomised trial**. *BJOG Int J Obstet Gy* 2016, **123**(2):233-243.
5. Dodd JM: **Dietary and lifestyle advice for pregnant women who are overweight or obese: the LIMIT randomized trial**. *Ann Nutr Metab* 2014, **64**(3-4):197-202.
6. Dodd JM, Kannieappan LM, Grivell RM, Deussen AR, Moran LJ, Yelland LN, Owens JA: **Effects of an antenatal dietary intervention on maternal anthropometric measures in pregnant women with obesity**. *Obesity* 2015, **23**(8):1555-1562.
7. Dodd JM, Turnbull D, McPhee AJ, Deussen AR, Grivell RM, Yelland LN, Crowther CA, Wittert G, Owens JA, Robinson JS: **Antenatal lifestyle advice for women who are overweight or obese: LIMIT randomised trial**. *BMJ* 2014, **348**.
8. Dodd JM, Turnbull DA, McPhee AJ, Wittert G, Crowther CA, Robinson JS: **Limiting weight gain in overweight and obese women during pregnancy to improve health outcomes: the LIMIT randomised controlled trial**. *BMC Pregnancy Childbirth* 2011, **11**:79.
9. Eiben G, Lissner L: **Health Hunters - An intervention to prevent overweight and obesity in young high-risk women**. *Int J Gynaecol Obstet* 2006, **30**(4):691-696.
10. Farajzadegan Z, Pozveh ZA: **The design of maternal centered life-style modification program for weight gain management during pregnancy - A study protocol**. *J Res Med Sci* 2013, **18**(8):683-687.
11. Ferrara A, Hedderson MM, Brown SD, Albright CL, Ehrlich SF, Tsai AL, Caan BJ, Sternfeld B, Gordon NP, Schmittdiel JA *et al*: **The Comparative Effectiveness of Diabetes Prevention Strategies to Reduce Postpartum Weight Retention in Women With Gestational Diabetes Mellitus: The Gestational Diabetes' Effects on Moms (GEM) Cluster Randomized Controlled Trial**. *Diabetes Care* 2016, **39**(1):65-74.
12. Foley P, Levine E, Askew S, Puleo E, Whiteley J, Batch B, Heil D, Dix D, Lett V, Lanpher M *et al*: **Weight gain prevention among black women in the rural community health center setting: the Shape Program**. *BMC Public Health* 2012, **12**(1):305-305.
13. Gesell SB, Katula JA, Strickland C, Vitolins MZ: **Feasibility and Initial Efficacy Evaluation of a Community-Based Cognitive-Behavioral Lifestyle Intervention to Prevent Excessive Weight Gain During Pregnancy in Latina Women**. In: *Matern Child Health J* vol. 19; 2015: 1842-1852.
14. Gilmore LA, Klempel MC, Martin CK, Myers CA, Burton JH, Sutton EF, Redman LM: **Personalized Mobile Health Intervention for Health and Weight Loss in Postpartum Women Receiving Women, Infants, and Children Benefit: A Randomized Controlled Pilot Study**. *J Womens Health (Larchmt)* 2017, **26**(7):719-727.
15. Graham ML, Strawderman MS, Demment M, Olson CM: **Does usage of an eHealth intervention reduce the risk of excessive gestational weight gain? Secondary analysis from a randomized controlled trial**. *J Med Internet Res* 2017, **19**(1).
16. Guelinckx I, Devlieger R, Mullie P, Vansant G: **Effect of lifestyle intervention on dietary habits, physical activity, and gestational weight gain in obese pregnant women: a randomized controlled trial**. *Am J Clin Nutr* 2010, **91**(2):373-380.
17. Haakstad LA, Bo K: **Effect of regular exercise on prevention of excessive weight gain in pregnancy: A randomised controlled trial**. *Eur J Contracept Reprod Health Care* 2011, **16**(2):116-125.
18. Haby K, Glantz A, Hanas R, Premberg A: **Mighty Mums - An antenatal health care intervention can reduce gestational weight gain in women with obesity**. *Midwifery* 2015, **31**(7):685-692.
19. Haire-Joshu DL, Schwarz CD, Peskoe SB, Budd EL, Brownson RC, Joshu CE: **A group randomized controlled trail integrating obesity prevention and control for postpartum adolescents in a home visiting program**. *Int J Behav Nutr Phy* 2015, **12**:88.
20. Harden SM, Beauchamp MR, Pitts BH, Nault EM, Davy BM, You W, Weiss P, Estabrooks PA: **Group-based lifestyle sessions for gestational weight gain management: a mixed method approach**. *Am J Health Behav* 2014, **38**(4):560-569.
21. Harrison CL, Lombard CB, Strauss BJ, Teede HJ: **Optimizing healthy gestational weight gain in women at high risk of gestational diabetes: A randomized controlled trial**. *Obesity* 2013, **21**(5):904-909.
22. Harrison CL, Lombard CB, Teede HJ: **Limiting postpartum weight retention through early antenatal intervention: The HeLP-her randomised controlled trial**. *Int J Behav Nutr Phy* 2014, **11**:134.
23. Harrison CL, Teede HJ, Lombard CB: **How effective is self-weighing in the setting of a lifestyle intervention to reduce gestational weight gain and postpartum weight retention?** *Aust N Z J Obstet Gynaecol* 2014, **54**(4):382-385.
24. Hawkins M, Hosker M, Marcus BH, Rosal MC, Braun B, Stanek EJ, 3rd, Markenson G, Chasan-Taber L: **A pregnancy lifestyle intervention to prevent gestational diabetes risk factors in overweight Hispanic women: a feasibility randomized controlled trial**. *Diabet Med* 2015, **32**(1):108-115.
25. Herring SJ, Cruice JF, Bennett GG, Darden N, Wallen JJ, Rose MZ, Davey A, Foster GD: **Intervening during and after pregnancy to prevent weight retention among African American women**. *Preventive Medicine Reports* 2017, **7**:119-123.
26. Herring SJ, Cruice JF, Bennett GG, Rose MZ, Davey A, Foster GD: **Preventing excessive gestational weight gain among African American women: A randomized clinical trial**. *Obesity* 2016, **24**(1):30-36.
27. Herring SJ, Cruice JF, Bennett GG, Davey A, Foster GD: **Using technology to promote postpartum weight loss in urban, low-income mothers: A pilot randomized controlled trial**. *J Nutr Educ Behav* 2014, **46**(6):610-615.

28. Huang TT, Yeh CY, Tsai YC: **A diet and physical activity intervention for preventing weight retention among Taiwanese childbearing women: a randomised controlled trial**. *Midwifery* 2011, **27**(2):257-264.
29. Hui A, Back L, Ludwig S, Gardiner P, Sevenhuysen G, Dean H, Sellers E, McGavock J, Morris M, Bruce S *et al*: **Lifestyle intervention on diet and exercise reduced excessive gestational weight gain in pregnant women under a randomised controlled trial**. *BJOG : an international journal of obstetrics and gynaecology* 2012, **119**(1):70-77.
30. Hui AL, Back L, Ludwig S, Gardiner P, Sevenhuysen G, Dean HJ, Sellers E, McGavock J, Morris M, Jiang D *et al*: **Effects of lifestyle intervention on dietary intake, physical activity level, and gestational weight gain in pregnant women with different pre-pregnancy Body Mass Index in a randomized control trial**. *BMC Pregnancy Childbirth* 2014, **14**:331.
31. Huseinovic E, Bertz F, Agelii ML, Johansson EH, Winkvist A, Brekke HK: **Effectiveness of a weight loss intervention in postpartum women: Results from a randomized controlled trial in primary health care**. *Am J Clin Nutr* 2016, **104**(2):362-370.
32. Jackson RA, Stotland NE, Caughey AB, Gerbert B: **Improving diet and exercise in pregnancy with Video Doctor counseling: A randomized trial**. *Patient Educ Couns* 2011, **83**(2):203-209.
33. Jeffries K, Shub A, Walker SP, Hiscock R, Permezel M: **Reducing excessive weight gain in pregnancy: a randomised controlled trial**. *Med J Aust* 2009, **191**(8):429-433.
34. Jing W, Huang Y, Liu X, Luo B, Yang Y, Liao S: **The effect of a personalized intervention on weight gain and physical activity among pregnant women in China**. *Int J Gynaecol Obstet* 2015, **129**(2):138-141.
35. John E, Cassidy DM, Playle R, Jewell K, Cohen D, Duncan D, Newcombe RG, Busse M, Owen-Jones E, Williams N *et al*: **Healthy eating and lifestyle in pregnancy (HELP): a protocol for a cluster randomised trial to evaluate the effectiveness of a weight management intervention in pregnancy**. *BMC Public Health* 2014, **14**:439.
36. Keller C, Ainsworth B, Records K, Todd M, Belyea M, Vega-Lopez S, Permana P, Coonrod D, Nagle-Williams A: **A comparison of a social support physical activity intervention in weight management among post-partum Latinas**. *BMC Public Health* 2014, **14**:971.
37. Kinnunen TI, Raitanen J, Aittasalo M, Luoto R: **Preventing excessive gestational weight gain-a secondary analysis of a cluster-randomised controlled trial**. *Eur J Clin Nutr* 2012, **66**(12):1344-1350.
38. Klem ML, Viteri JE, Wing RR: **Primary prevention of weight gain for women aged 25-34: The acceptability of treatment formats**. *Int J Obes* 2000, **24**(2):219-225.
39. Koivusalo SB, Rono K, Klemetti MM, Roine RP, Lindstrom J, Erkkola M, Kaaja RJ, Poyhonen-Alho M, Tiitinen A, Huvinen E *et al*: **Gestational Diabetes Mellitus Can Be Prevented by Lifestyle Intervention: The Finnish Gestational Diabetes Prevention Study (RADIEL): A Randomized Controlled Trial**. *Diab Care* 2016, **39**(1):24-30.
40. Lim SS, Norman RJ, Clifton PM, Noakes M: **Psychological effects of prescriptive vs general lifestyle advice for weight loss in young women**. *J Am Diet Assoc* 2009, **109**(11):1917-1921.
41. Martin J, MacDonald-Wicks L, Hure A, Smith R, Collins CE: **Reducing postpartum weight retention and improving breastfeeding outcomes in overweight women: a pilot randomised controlled trial**. *Nutrients* 2015, **7**(3):1464-1479.
42. McCarthy EA, Walker SP, Ugoni A, Lappas M, Leong O, Shub A: **Self-weighing and simple dietary advice for overweight and obese pregnant women to reduce obstetric complications without impact on quality of life: a randomised controlled trial**. *BJOG : an international journal of obstetrics and gynaecology* 2016, **123**(6):965-973.
43. Mutsaerts M: **Randomized trial of a lifestyle program in obese infertile women**. In: *Ned Tijdschr Geneeskd.* vol. 161; 2017.
44. van Dammen L, Wekker V, Van Oers AM, Mutsaerts MAQ, Painter RC, Zwinderman AH, Groen H, van de Beek C, Muller Kobold AC, Kuchenbecker WKH *et al*: **Effect of a lifestyle intervention in obese infertile women on cardiometabolic health and quality of life: A randomized controlled trial**. *PLoS ONE* 2018, **13 (1)** (e0190662).
45. Mutsaerts MA, Groen H, ter Bogt NC, Bolster JH, Land JA, Bemelmans WJ, Kuchenbecker WK, Hompes PG, Macklon NS, Stolk RP *et al*: **The LIFESTYLE study: costs and effects of a structured lifestyle program in overweight and obese subfertile women to reduce the need for fertility treatment and improve reproductive outcome. A randomised controlled trial**. *BMC Women's Health* 2010, **10**:22.
46. Nicklas JM, Zera CA, England LJ, Rosner BA, Horton E, Levkoff SE, Seely EW: **A web-based lifestyle intervention for women with recent gestational diabetes mellitus: a randomized controlled trial**. *Obstet Gynecol* 2014, **124**(3):563-570.
47. Østbye T, Krause KM, Lovelady CA, Morey MC, Bastian LA, Peterson BL, Swamy GK, Brouwer RJN, McBride CM: **Active Mothers Postpartum. A Randomized Controlled Weight-Loss Intervention Trial**. *Am J Prev Med* 2009, **37**(3):173-180.
48. Peccei A, Blake-Lamb T, Rahilly D, Hatoum I, Bryant A: **Intensive Prenatal Nutrition Counseling in a Community Health Setting: A Randomized Controlled Trial**. *Obstet Gynecol* 2017, **130**(2):423-432.
49. Petrella E, Malavolti M, Bertarini V, Pignatti L, Neri I, Battistini NC, Facchinetti F: **Gestational weight gain in overweight and obese women enrolled in a healthy lifestyle and eating habits program**. *J Matern Fetal Neonatal Med* 2014, **27**(13):1348-1352.
50. Phelan S, Brannen A, Erickson K, Diamond M, Schaffner A, Muñoz-Christian K, Stewart A, Sanchez T, Rodriguez VC, Ramos DI *et al*: **'Fit Moms/Mamás Activas' internet-based weight control program with group support to reduce postpartum weight retention in low-income women: study protocol for a randomized controlled trial**. In: *Trials.* vol. 16; 2015: 59.
51. Phelan S, Hagobian T, Brannen A, Hatley KE, Schaffner A, Muñoz-Christian K, Tate DF: **Effect of an Internet-Based Program on Weight Loss for Low-Income Postpartum Women: a Randomized Clinical Trial**. In: *JAMA.* vol. 317; 2017: 2381-2391.
52. Phelan S, Phipps MG, Abrams B, Darroch F, Schaffner A, Wing RR: **Randomized trial of a behavioral intervention to prevent excessive gestational weight gain: the Fit for Delivery Study**. *Am J Clin Nutr* 2011, **93**(4):772-779.
53. Phelan S, Phipps MG, Abrams B, Darroch F, Grantham K, Schaffner A, Wing RR: **Does behavioral intervention in pregnancy reduce postpartum weight retention? Twelve-month outcomes of the Fit for Delivery randomized trial**. *Am J Clin Nutr* 2014, **99**(2):302-311.
54. Pollak KI, Alexander SC, Bennett G, Lyna P, Coffman CJ, Bilheimer A, Farrell D, Bodner ME, Swamy GK, Ostbye T: **Weight-related SMS texts promoting appropriate pregnancy weight gain: A pilot study**. *Patient Educ Couns* 2014, **97**(2):256-260.
55. Polley B, Wing R, Sims C: **Randomized controlled trial to prevent excessive weight gain in pregnant women**. *Int J Obes* 2002, **26**(11):1494-1502.
56. Quinlivan JA, Lam LT, Fisher J: **A randomised trial of a four-step multidisciplinary approach to the antenatal care of obese pregnant women**. *Aust N Z J Obstet Gynaecol* 2011, **51**(2):141-146.
57. Rauh K, Gabriel E, Kerschbaum E, Schuster T, Kries R, Amann-Gassner U, Hauner H: **Safety and efficacy of a lifestyle intervention for pregnant women to prevent excessive maternal weight gain: a cluster-randomized controlled trial**. In: *BMC Pregnancy Childbirth.* vol. 13; 2013.
58. Rauh K, Gunther J, Kunath J, Stecher L, Hauner H: **Lifestyle intervention to prevent excessive maternal weight gain: mother and infant follow-up at 12 months postpartum**. *BMC Pregnancy Childbirth* 2015, **15**:265.
59. Rauh K, Kunath J, Rosenfeld E, Kick L, Ulm K, Hauner H: **Healthy living in pregnancy: a cluster-randomized controlled trial to prevent excessive gestational weight gain - rationale and design of the GeliS study**. *BMC Pregnancy Childbirth* 2014, **14**:119.
60. Renault KM, Carlsen EM, Haedersdal S, Nilas L, Secher NJ, Eugen-Olsen J, Cortes D, Olsen SF, Halldorsson TI, Norgaard K: **Impact of lifestyle intervention for obese women during pregnancy on maternal metabolic and inflammatory markers**. *Int J Obes* 2017, **41**(4):598-605.
61. Ronnberg AK, Ostlund I, Fadl H, Gottvall T, Nilsson K: **Intervention during pregnancy to reduce excessive gestational weight gain - A randomised controlled trial**. In: *BJOG : an international journal of obstetrics and gynaecology.* vol. 122; 2015: 537-544.
62. Ronnberg A, Hanson U, Ostlund I, Nilsson K: **Effects on postpartum weight retention after antenatal lifestyle intervention - a secondary analysis of a randomized controlled trial**. *Acta Obstet Gynecol Scand* 2016, **95**(9):999-1007.
63. Sagedal LR, Overby NC, Bere E, Torstveit MK, Lohne-Seiler H, Smastuen M, Hillesund ER, Henriksen T, Vistad I: **Lifestyle intervention to limit gestational weight gain: the Norwegian Fit for Delivery randomised controlled trial**. *BJOG Int J Obstet Gy* 2017, **124**(1):97-109.
64. Sagedal LR, Sanda B, Overby NC, Bere E, Torstveit MK, Lohne-Seiler H, Hillesund ER, Pripp AH, Henriksen T, Vistad I: **The effect of prenatal lifestyle intervention on weight retention 12 months postpartum: results of the Norwegian Fit for Delivery randomised controlled trial**. *BJOG : an international journal of obstetrics and gynaecology* 2017, **124**(1):111-121.
65. Sanda B, Vistad I, Sagedal LR, Haakstad LAH, Lohne-Seiler H, Torstveit MK: **Effect of a prenatal lifestyle intervention on physical activity level in late pregnancy and the first year postpartum**. *PLoS ONE [Electronic Resource]* 2017, **12**(11):e0188102.
66. Skouteris H, McPhie S, Hill B, McCabe M, Milgrom J, Kent B, Bruce L, Herring S, Gale J, Mihalopoulos C *et al*: **Health coaching to prevent excessive gestational weight gain: A randomized-controlled trial**. *Br J Health Psychol* 2016, **21**(1):31-51.
67. Smith K, Lanningham-Foster L, Welch A, Campbell C: **Web-Based Behavioral Intervention Increases Maternal Exercise but Does Not Prevent Excessive Gestational Weight Gain in Previously Sedentary Women**. *J Phys Act Health* 2016, **13**(6):587-593.
68. Stendell-Hollis NR, Thompson PA, West JL, Wertheim BC, Thomson CA: **A comparison of Mediterranean-style and MyPyramid diets on weight loss and inflammatory biomarkers in postpartum breastfeeding women**. *J Womens Health (Larchmt)* 2013, **22**(1):48-57.
69. Szmeja MA, Cramp C, Grivell RM, Deussen AR, Yelland LN, Dodd JM: **Use of a DVD to provide dietary and lifestyle information to pregnant women who are overweight or obese: a nested randomised trial**. *BMC Pregnancy Childbirth* 2014, **14**:409.

70. Tawfik MY: **The Impact of Health Education Intervention for Prevention and Early Detection of Type 2 Diabetes in Women with Gestational Diabetes**. *J Community Health* 2017, **42**(3):500-510.
71. Thomson JL, Tussing-Humphreys LM, Goodman MH: **Delta Healthy Sprouts: a randomized comparative effectiveness trial to promote maternal weight control and reduce childhood obesity in the Mississippi Delta**. *Contemp Clin Trials* 2014, **38**(1):82-91.
72. Thomson JL, Tussing-Humphreys LM, Goodman MH, Olender SE: **Gestational Weight Gain: results from the Delta Healthy Sprouts Comparative Impact Trial**. In: *Journal of Pregnancy.* vol. 2016; 2016: 5703607.
73. Thornton YS: **Preventing excessive weight gain during pregnancy through dietary and lifestyle counseling: a randomized controlled trial**. *Obstet Gynecol* 2009, **114**(1):173; author reply 173-174.
74. Vesco KK, Karanja N, King JC, Gillman MW, Perrin N, McEvoy C, Eckhardt C, Smith KS, Stevens VJ: **Healthy Moms, a randomized trial to promote and evaluate weight maintenance among obese pregnant women: study design and rationale**. *Contemp Clin Trials* 2012, **33**(4):777-785.
75. Vesco KK, Karanja N, King JC, Gillman MW, Leo MC, Perrin N, McEvoy CT, Eckhardt CL, Smith K, Stevens VJ: **Efficacy of a group-based dietary intervention for limiting gestational weight gain among obese women: A randomized trial**. *Obesity* 2014, **22**(9):1989-1996.
76. Vesco KK, Leo MC, Karanja N, Gillman MW, McEvoy CT, King JC, Eckhardt CL, Smith KS, Perrin N, Stevens VJ: **One-year postpartum outcomes following a weight management intervention in pregnant women with obesity**. *Obesity* 2016, **24**(10):2042-2049.
77. Vinter CA, Jensen DM, Ovesen P, Beck-Nielsen H, Jorgensen JS: **The LiP (Lifestyle in Pregnancy) study: a randomized controlled trial of lifestyle intervention in 360 obese pregnant women**. *Diab Care* 2011, **34**(12):2502-2507.
78. Vinter C, Jensen D, Ovesen P, Beck-Nielsen H, Lamont R, Jorgensen J: **Postpartum weight retention and breastfeeding among obese women from the LiP (Lifestyle in Pregnancy) Study**. *Acta Obstet Gynecol Scand* 2012, **159)**:141-142.
79. Vinter CA, Jensen DM, Ovesen P, Beck-Nielsen H, Tanvig M, Lamont RF, Jorgensen JS: **Postpartum weight retention and breastfeeding among obese women from the randomized controlled Lifestyle in Pregnancy (LiP) trial**. *Acta Obstet Gynecol Scand* 2014, **93**(8):794-801.
80. Wilkinson SA, Pligt P, Gibbons KS, McIntyre HD: **Trial for Reducing Weight Retention in New Mums: a randomised controlled trial evaluating a low intensity, postpartum weight management programme**. In: *J Hum Nutr Diet.* vol. 28 Suppl 1; 2015: 15-28.
81. Willcox J, Wilkinson S, Lappas M, Ball K, Crawford D, McCarthy E, Fjeldsoe B, Whittaker R, Maddison R, Campbell K: **A mobile health intervention promoting healthy gestational weight gain for women entering pregnancy at a high body mass index: the txt4two pilot randomised controlled trial**. In: *BJOG : an international journal of obstetrics and gynaecology.* vol. (no pagination); 2017.
82. Wolff S, Legarth J, Vangsgaard K, Toubro S, Astrup A: **A randomized trial of the effects of dietary counseling on gestational weight gain and glucose metabolism in obese pregnant women**. *Int J Obes* 2008, **32**(3):495-501.
83. Abdel-Aziz SB, Hegazy IS, Mohamed DA, Abu El Kasem MMA, Hagag SS: **Effect of dietary counseling on preventing excessive weight gain during pregnancy**. *Public Health* 2018, **154**:172-181.
84. Adamo KB, Ferraro ZM, Goldfield G, Keely E, Stacey D, Hadjiyannakis S, Jean-Philippe S, Walker M, Barrowman NJ: **The Maternal Obesity Management (MOM) Trial Protocol: a lifestyle intervention during pregnancy to minimize downstream obesity**. *Contemp Clin Trials* 2013, **35**(1):87-96.
85. Althuizen E, Poppel MNM, Seidell JC, Wijden C, Mechelen W: **Design of the New Life(style) study: a randomised controlled trial to optimise maternal weight development during pregnancy**. In: *BMC Public Health.* vol. 6; 2006: 168.
86. Althuizen E, Van Der Wijden CL, Van Mechelen W, Seidell JC, Van Poppel MNM: **The effect of a counselling intervention on weight changes during and after pregnancy: A randomised trial**. *BJOG Int J Obstet Gy* 2013, **120**(1):92-99.
87. Ames GE, Perri MG, Fox LD, Fallon EA, De Braganza N, Murawski ME, Pafumi L, Hausenblas HA: **Changing weight-loss expectations: A randomized pilot study**. *Eating Behaviors* 2005, **6**(3):259-269.
88. Asbee SM, Jenkins TR, Butler JR, White J, Elliot M, Rutledge A: **Preventing excessive weight gain during pregnancy through dietary and lifestyle counseling: a randomized controlled trial**. *Obstet Gynecol* 2009, **113**(2, Part 1):305-312.
89. Asci O, Rathfisch G: **Effect of lifestyle interventions of pregnant women on their dietary habits, lifestyle behaviors, and weight gain: a randomized controlled trial**. *J Health Popul Nutr* 2016, **35**:7.
90. Bechtel-Blackwell DA: **Computer-assisted self-interview and nutrition education in pregnant teens**. *Clin Nurs Res* 2002, **11**(4):450-462.
91. Bennett GG, Foley P, Levine E, Whiteley J, Askew S, Steinberg DM, Batch B, Greaney ML, Miranda H, Wroth TH *et al*: **Behavioral treatment for weight gain prevention among black women in primary care practice: a randomized clinical trial**. In: *JAMA Intern Med.* vol. 173; 2013: 1770-1777.
92. Berry D, Colindres M, Sanchez-Lugo L, Sanchez M, Neal M, Smith-Miller C: **Adapting, Feasibility Testing, and Pilot Testing a Weight Management Intervention for Recently Immigrated Spanish-Speaking Women and Their 2- to 4-Year-Old Children**. *Hispanic Health Care International* 2011, **9**(4):186-193.
93. Berry D, Verbiest S, Hall EG, Dawson I, Norton D, Willis S, McDonald K, Stuebe A: **A postpartum community-based weight management intervention designed for low-income women: Feasibility and initial efficacy testing**. *J Natl Black Nurses Assoc* 2015, **26**(1):29-39.
94. Bertz F, Brekke HK, Ellegard L, Rasmussen KM, Wennergren M, Winkvist A: **Diet and exercise weight-loss trial in lactating overweight and obese women**. *Am J Clin Nutr* 2012, **96**(4):698-705.
95. Brekke HK, Bertz F, Rasmussen KM, Bosaeus I, Ellegard L, Winkvist A: **Diet and exercise interventions among overweight and obese lactating women: randomized trial of effects on cardiovascular risk factors**. *PLoS ONE [Electronic Resource]* 2014, **9**(2):e88250.
96. Bertz F, Winkvist A, Brekke HK: **Sustainable weight loss among overweight and obese lactating women is achieved with an energy-reduced diet in line with dietary recommendations: results from the LEVA randomized controlled trial**. *J Acad Nutr Diet* 2015, **115**(1):78-86.
97. Huseinovic E, Winkvist A, Bertz F, Brekke HK: **Changes in food choice during a successful weight loss trial in overweight and obese postpartum women**. *Obesity* 2014, **22**(12):2517-2523.
98. Bogaerts A, Devlieger R, Nuyts E, Witters I, Gyselaers W, Van den Bergh B: **Effects of lifestyle intervention in obese pregnant women on gestational weight gain and mental health: a randomized controlled trial**. *Int J Gynaecol Obstet* 2013, **37**(6):814.
99. Brownfoot FC, Davey MA, Kornman L: **Routine weighing to reduce excessive antenatal weight gain: a randomised controlled trial**. *BJOG : an international journal of obstetrics and gynaecology* 2016, **123**(2):254-261.
100. Chang MW, Nitzke S, Brown R: **Design and outcomes of a Mothers In Motion behavioral intervention pilot study**. *J Nutr Educ Behav* 2010, **42**(3 Suppl):S11-21.
101. Chang MW, Nitzke S, Brown R, Egan MJB, Bendekgey CM, Buist D: **Recruitment challenges and enrollment observations from a community based intervention (Mothers In Motion) for low-income overweight and obese women**. *Contemp Clin Trials* 2017, **5**:26-33.
102. Chang MW, Brown R, Nitzke S: **Results and lessons learned from a prevention of weight gain program for low-income overweight and obese young mothers: Mothers In Motion**. *BMC Public Health* 2017, **17**(1):182.
103. Chasan-Taber L, Marcus BH, Rosal MC, Tucker KL, Hartman SJ, Pekow P, Stanek E, Braun B, Solomon CG, Manson JAE *et al*: **Proyecto Mama: A lifestyle intervention in overweight and obese Hispanic women: A randomised controlled trial - study protocol**. *BMC Pregnancy Childbirth* 2015, **15 (1)**(157).
104. Clements V, Leung K, Khanal S, Raymond J, Maxwell M, Rissel C: **Pragmatic cluster randomised trial of a free telephone-based health coaching program to support women in managing weight gain during pregnancy: the Get Healthy in Pregnancy Trial**. *BMC Health Serv Res* 2016, **16**:454.
105. Colleran HL, Lovelady CA: **Use of MyPyramid Menu Planner for Moms in a weight-loss intervention during lactation**. *J Acad Nutr Diet* 2012, **112**(4):553-558.
106. Craigie AM, Macleod M, Barton KL, Treweek S, Anderson AS: **Supporting postpartum weight loss in women living in deprived communities: design implications for a randomised control trial**. *Eur J Clin Nutr* 2011, **65**(8):952-958.
107. Hui AL, Ludwig S, Gardiner P, Sevenhuysen G, Murray R, Morris M, Shen GX: **Community-based Exercise and Dietary Intervention During Pregnancy:A Pilot Study**. *Can J Diab* 2006, **30**(2):1-7.
108. Krummel D, Semmens E, MacBride AM, Fisher B: **Lessons learned from the mothers' overweight management study in 4 West Virginia WIC offices**. *J Nutr Educ Behav* 2010, **42**(3 Suppl):S52-S58.
109. Leermakers EA, Anglin K, Wing RR: **Reducing postpartum weight retention through a correspondence intervention**. *International journal of obesity and related metabolic disorders : journal of the International Association for the Study of Obesity* 1998, **22**(11):1103-1109.
110. O'Toole ML, Sawicki MA, Artal R: **Structured diet and physical activity prevent postpartum weight retention**. *J Womens Health (Larchmt)* 2003, **12**(10):991-998.
111. Nagle C, Skouteris H, Hotchin A, Bruce L, Patterson D, Teale G: **Continuity of midwifery care and gestational weight gain in obese women: a randomised controlled trial**. *BMC Public Health* 2011, **11**:174.

112. LeBlanc ES, Vesco KK, Funk KL, Karanja N, Smith N, Stevens VJ: **Prepare, a randomized trial to promote and evaluate weight loss among overweight and obese women planning pregnancy: Study design and rationale**. *Contemp Clin Trials* 2016, **49**:174-180.
113. Brown MJ, Sinclair M, Liddle D, Hill AJ, Madden E, Stockdale J: **A systematic review investigating healthy lifestyle interventions incorporating goal setting strategies for preventing excess gestational weight gain**. *PLoS ONE [Electronic Resource]* 2012, **7**(7):e39503.
114. Kuhlmann AK, Dietz PM, Galavotti C, England LJ: **Weight-management interventions for pregnant or postpartum women**. *Am J Prev Med* 2008, **34**(6):523-528.
115. Quinlivan JA, Julania S, Lam L: **Antenatal dietary interventions in obese pregnant women to restrict gestational weight gain to Institute of Medicine recommendations: a meta-analysis**. *Obstet Gynecol* 2011, **118**(6):1395-1401.
